# Asymmetric introgression reveals the genetic architecture of a plumage trait

**DOI:** 10.1038/s41467-021-21340-y

**Published:** 2021-02-15

**Authors:** Georgy A. Semenov, Ethan Linck, Erik D. Enbody, Rebecca B. Harris, David R. Khaydarov, Per Alström, Leif Andersson, Scott A. Taylor

**Affiliations:** 1 Ecology and Evolutionary Biology, University of Colorado, Boulder, CO USA; 2 UNM Biology, University of New Mexico, Albuquerque, NM Mexico; 3 Department of Medical Biochemistry and Microbiology, Uppsala University, Uppsala, Sweden; 4 Adaptive Biotechnologies, E Seattle, WA USA; 5 School 171, Moscow, Russian Federation; 6 Animal Ecology, Department of Ecology and Genetics, Evolutionary Biology Centre, Uppsala University, Uppsala, Sweden; 7 Key Laboratory of Zoological Systematics and Evolution, Institute of Zoology, Chinese Academy of Sciences, Beijing, China; 8 Department of Veterinary Integrative Biosciences, College of Veterinary Medicine and Biomedical Sciences, Texas A&M University, College Station, TX USA; 9 Department of Animal Breeding and Genetics, Swedish University of Agricultural Sciences, Uppsala, Sweden

**Keywords:** Evolutionary genetics, Evolutionary biology

## Abstract

Genome-wide variation in introgression rates across hybrid zones offers a powerful opportunity for studying population differentiation. One poorly understood pattern of introgression is the geographic displacement of a trait implicated in lineage divergence from genome-wide population boundaries. While difficult to interpret, this pattern can facilitate the dissection of trait genetic architecture because traits become uncoupled from their ancestral genomic background. We studied an example of trait displacement generated by the introgression of head plumage coloration from personata to alba subspecies of the white wagtail. A previous study of their hybrid zone in Siberia revealed that the geographic transition in this sexual signal that mediates assortative mating was offset from other traits and genetic markers. Here we show that head plumage is associated with two small genetic regions. Despite having a simple genetic architecture, head plumage inheritance is consistent with partial dominance and epistasis, which could contribute to its asymmetric introgression.

## Abstract (web summary)

Hybrid zones are windows into the evolutionary process. Semenov et al. find that the head plumage differences between white wagtail subspecies have a simple genetic basis involving two small genetic regions, in which partially dominant and epistatic interactions help to explain how this sexual signal has become decoupled from other plumage traits.

## Introduction

Hybrid zones have long been recognized as windows into the evolutionary process^1–5^. If hybrids are not completely sterile, hybridization will often result in backcrossing and a flux of foreign alleles into parental populations, i.e. introgression. Introgression rates vary substantially across the genome due to local differences in the strength of selection, frequency of recombination, and the interplay between selection and recombination along chromosomes^6–9^. Loci directly targeted by divergent selection (or closely linked to its targets) will show particularly reduced introgression rates compared to the genome-wide average (assuming minimal coupling and a lack of strong barriers to gene flow), aiding in the detection of loci associated with population divergence^10–12^.

An intriguing observation, now recorded in several study systems, is the geographic displacement of a trait under selection away from the majority of genetic markers^13–16^. This pattern poses an evolutionary quandary. In a given hybrid zone, we expect loci underpinning traits under divergent selection to show narrower clines than those of neutral loci elsewhere in the genome. Yet, if these divergently selected loci act as “barriers” promoting incipient speciation, we also expect their cline centers to be co-located with the cline centers of neutral loci varying across species. Asymmetric sexual or natural selection is often invoked to explain this pattern. Indeed, simulation studies suggest that differences in allele fitness and selection strength^17,18^, as well as epistasis^19^, and deviation from codominant genetic inheritance of selected phenotypes^17^ can contribute to asymmetric introgression. However, there exist few empirical examples in the literature due to the difficulty of dissecting the genetic architecture of phenotypic traits in natural study systems—data crucial for distinguishing among these potential mechanisms. Furthermore, the connection between trait genetic architecture and asymmetric introgression under a scenario of positive assortative mating—commonly found in the hybrid zones—remains poorly understood^19^.

**Fig. 1:**
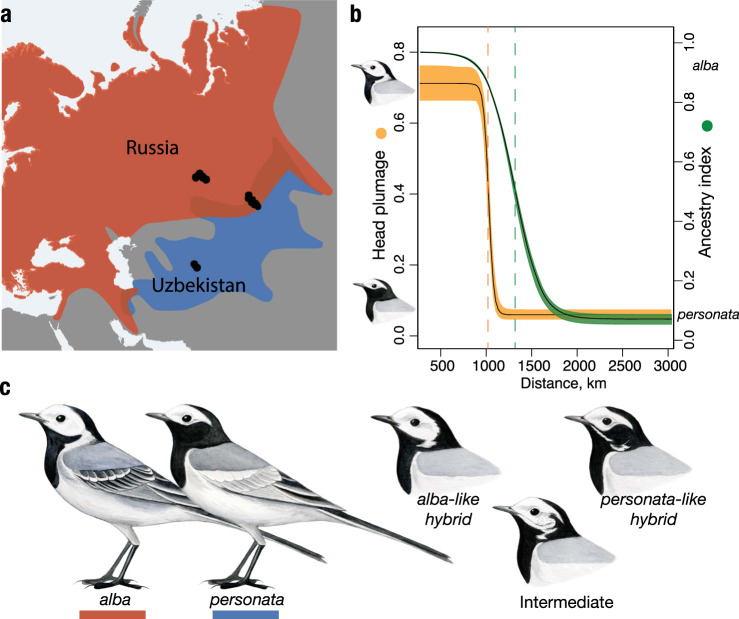
Distribution of *alb*a and *personata* subspecies of the white wagtail and their hybridization patterns. **a** Geographic distribution and sampling localities used in the present study (black dots). Areas of red (*alba*) and blue (*personata*) color overlap are their hybrid zones. **b** Geographic clines in head plumage coloration (orange) and genetic ancestry (green) for a 3000 km transect across the *alba* and *personata* hybrid zone in central Siberia (redrawn from ref. ^16^). Dashed lines show cline centers, shading is a 95% CI. **c** Plumage differences between *alba* and *personata*, and intermediate variants of head plumage observed in their hybrid zone. The allopatric *alba* and *personata* samples originate from terminal parts of the transect in Russia and Uzbekistan, respectively. The individuals we refer to as sympatric *alba* and *personata* and all intermediate head plumage phenotypes, were sampled in the area of range overlap (see Supplementary Table 1 for sampling details). Drawings are courtesy of Jillian Ditner.

The white wagtail (*Motacilla alba*) is a small passerine bird with nine commonly recognized subspecies distributed across most of Eurasia, North Africa, and northwestern North America^20,21^. Historically, white wagtails likely bred on the banks of rivers and other water bodies^22^; now they are a common synanthropic species in many parts of their range^20,23^. The subspecies *M. a. alba* and *M. a. personata* differ in a suite of phenotypic traits that distinguish allopatric populations (e.g., body size, head patterning, intensity of gray plumage on the back, and amount of white plumage on the wing) and hybridize in two geographically isolated regions in Iran and central Siberia^16,20^ (Fig. 1a). A previous study of phenotypic geographic clines in the Siberian hybrid zone revealed that only coloration of head and neck plumage showed a sharp, ~100-kilometer transition, while other phenotypic traits varied gradually^16^ (Fig. 1b). Further, the segregation pattern of head plumage recorded in the same study included only a few discrete intermediate categories (Fig. 1c), suggesting that head plumage is determined by few genes of large effect. The majority of individuals in the geographic area across which head plumage transitioned possessed either *alba* (33%) or *personata* (29%) head plumages, while the remaining individuals were 20% *alba*-like hybrids, 4% intermediates, and 14% *personata*-like hybrids (*n* = 104). Strong positive assortative mating by head plumage (i.e., the composition of social pairs deviated significantly from a scenario of random mating based on randomization statistical analysis^16^) and the observed predominance of parental phenotypes over hybrids are consistent with the maintenance of restricted introgression of head plumage by selection.

The apparent role for head plumage as a reproductive barrier is hard to reconcile with the advanced introgression of the melanic *personata* head phenotype into the *alba* genomic background^16^, revealed by a 323-kilometer displacement between the centers of the head plumage and genetic ancestry geographic clines (Fig. 1b). Many locations across the contact zone are characterized by individuals with the *personata* head phenotype, but genetic ancestry indistinguishable from allopatric *alba* populations^16^. This introgression pattern could be due to hybrid zone movement towards the range of the *alba* subspecies, where the head plumage cline represents the leading edge of the moving hybrid zone and the genetic ancestry cline is displaced due to genome-wide flux of selectively neutral alleles towards *personata*—a common finding in moving hybrid zones^24^. Alternatively, *personata* head plumage could be asymmetrically introgressing away from an otherwise spatially stable area of genome-wide transition. While multiple mechanisms, potentially involving selection, can be invoked to explain the pattern of asymmetric introgression, they are hard to disentangle without knowing the genetic loci that underlie the trait of interest. Beyond being a striking and unsolved evolutionary puzzle, asymmetric introgression of head plumage in wagtails also offers a rare opportunity to dissect the genetic basis of a phenotype mediating assortative mating due to its decoupling from the ancestral genomic background.

In this study, we utilize whole genome analysis of many individuals to investigate the genetic architecture of *alba* and *personata* head plumage differences. We show that head plumage is associated with only two small (150–200 kb in size) genomic regions on chromosomes 1A and 20, of which the latter contains the agouti signaling protein gene and appears to have the major phenotypic effect. Despite seemingly simple genetic architecture, head plumage inheritance patterns support a model of partial dominance and epistatic interactions between the two genomic regions. Our findings suggest that peculiarities of genetic architecture may contribute to asymmetric introgression of head plumage, therefore illuminating the connection between mechanisms of formation of reproductive barriers between nascent species and evolutionary dynamics of these barriers.

## Results

### Genetic architecture of plumage differences in wagtails

We sequenced whole genomes of allopatric *alba* (*n* = 10), allopatric *personata* (*n* = 10), and 62 individuals from the hybrid zone (18 phenotypic *alba* and 20 phenotypic *personata*, 11 *alba*-like hybrids, four strict intermediates, nine *personata*-like hybrids) at 5–7.5× depth of coverage (Fig. 1a, Supplementary Table 1, Supplementary Fig. 1), as well as one individual each from two outgroup species, Mekong wagtail *Motacilla samveasnae* and citrine wagtail *M. citreola*. Sequence reads were mapped to an eastern yellow wagtail *M. tschutschensis tschutschensis* draft reference genome (see the “Methods” section). After applying stringent filters (see the “Methods” section), we obtained ~10.6M SNPs for downstream analyses.

**Fig. 2:**
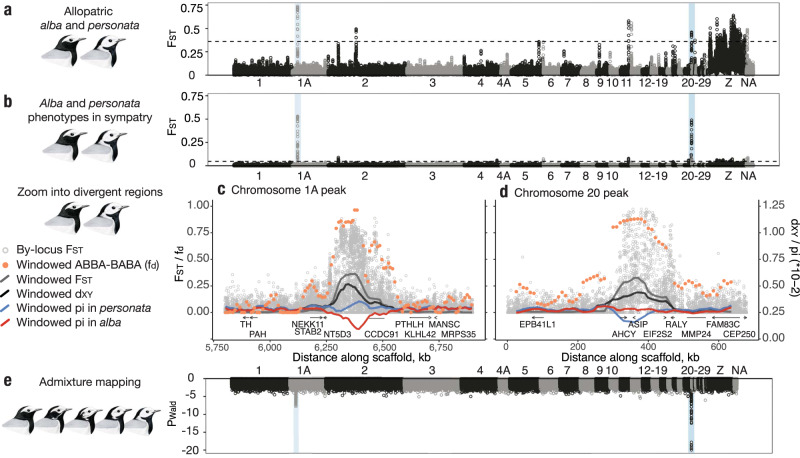
Whole-genome differentiation between *alba* and *personata* subspecies and the results of admixture mapping. **a**, **b** Show relative genomic differentiation (*F*_ST_) between remote allopatric populations and sympatric *alba* and *personata* head plumage phenotypes, respectively, estimated in 25 kb windows with 10 kb steps. Note that among multiple regions differentiated between allopatric populations, only two peaks on chromosomes 1A and 20 (highlighted in blue) remain distinct in sympatry. **c**, **d** Expand the highlighted regions on chromosomes 1A and 20. Gray circles are by-locus Weir-and-Cockerham *F*_ST_, light gray and black lines are window-based estimates of the relative (*F*_ST_) and absolute (*d*_*XY*_) differentiation, respectively. Red and blue lines are window-based estimates of nucleotide diversity in *alba* and *personata*, respectively. Orange dots are window-based ABBA–BABA (*f*_d_) indicating a fraction of shared ancestry between sympatric and allopatric *personata* (50 kb windows with 10 kb steps, see the “Methods” section for details). Below each plot are gene models with arrows indicating the direction of reading frame where it is known. Note that the divergent peak on chromosome 1A is mostly located in a non-coding region but includes the coiled-coil domain-containing protein 91 (*CCDC91*) gene. The peak on chromosome 20 overlaps three genes: adenosylhomocysteinase (*AHCY*), eukaryotic translation initiation factor 2 subunit beta (*EIF2S2*), and is centered around agouti signaling protein (*ASIP*). **e** Results of admixture mapping for head plumage. Each dot is a −log_10_-transformed *P*-value for rejecting a model assuming no association between variation in a trait and a genetic locus. Dashed lines on **a** and **b** are 0.995 quantiles.

Genomic differentiation between remote allopatric populations of *alba* and *personata* was low (weighted *F*_ST_ = 0.046), but sufficient to detect population structure (Supplementary Fig. 2). Overall, differences between subspecies were concentrated in numerous, mostly narrow, regions of high *F*_ST_ (Fig. 2a). Genomic differentiation between *alba* and *personata* head plumage phenotypes sampled within their hybrid zone was an order of magnitude lower (*F*_ST_ = 0.002), with only two strongly differentiated regions located on chromosomes 1A (~200 kb in size) and 20 (~150 kb in size) (Fig. 2b–d), and no genome-wide signal of population structure (Supplementary Fig. 2).

We performed admixture mapping using whole genomes of 62 individuals sampled within the hybrid zone to test for an association between individual SNPs and variation in head plumage. The only genomic regions with strong plumage associations coincided with peaks of genomic differentiation on chromosomes 1A and 20, with the region on chromosome 20 exhibiting the strongest signal (maximum −log_10_ *P* = 22.5, Fig. 2e) compared to 1A (maximum −log_10_ *P* = 8.1). Further, an analysis of genetic architecture of head plumage using a Bayesian sparse linear mixed model (BSLMM) indicated that 96.4 ± 3.6% of the phenotypic variance was explained by the complete set of ~11M SNPs, of which sparse effect terms (i.e., alleles with large phenotypic effect) accounted for 96.0 ± 4.0%. The average number of SNPs in the best model was 5.3 ± 2.6 loci, with up to 11.8% and 50.9% of variance explained by SNPs in divergent regions on chromosomes 1A and 20, respectively, all consistent with a simple genetic architecture of head plumage. To assess the predictive power of BSLMM, we used leave-one-out cross validation. Specifically, we iteratively excluded the phenotypic information from one individual and used the model fit for the remaining individuals to predict that phenotype based on its genotype (Supplementary Fig. 3). This analysis revealed high predictive power for sympatric parental phenotypes (adjusted *R*^2^ = 0.78) but rather moderate predictive power for hybrids (adjusted *R*^2^ = 0.54), suggesting that the relationship between phenotypic and genetic variation is non-linear to some extent and indicating potential deviations from additive and codominant inheritance models.

Local increases in relative (*F*_ST_) and absolute (*d*_*XY*_) divergence in the regions on chromosomes 1A and 20 (Fig. 2c, d) were consistent with selection maintaining genetic differences despite gene flow^25^. Further, an admixture proportion estimated in the context of ABBA–BABA (*f*_d_)^26^ revealed a strong excess of shared ancestry between allopatric and sympatric *personata* in both regions (Fig. 2c, d), supporting the hypothesis that the two genomic regions separating sympatric *personata* from *alba* have introgressed from allopatric *personata*. The peak on chromosome 1A was located in a highly homozygous (Supplementary Fig. 4) stretch of non-coding sequence between the genes 5′-nucleotidase domain-containing protein 3 (*NT5D3*) and coiled-coil domain-containing protein 91 (*CCDC91*) (Fig. 2c). The peak on chromosome 20 overlapped three genes (Fig. 2d) and was centered around agouti signaling protein (*ASIP*)—a well-studied gene regulating melanogenesis—with highly homozygous non-coding regions (Supplementary Fig. 4) occurring upstream and downstream of the ASIP reading frame in “masked” *personata*-like phenotypes (Supplementary Fig. 4).

**Fig. 3:**
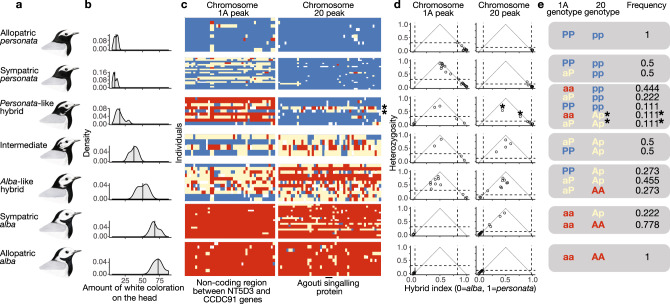
Genetic architecture and inheritance mechanism of head plumage differences in wagtails. **a** Typical phenotypes for each group. **b** Distribution of head plumage values estimated as a number of white pixels on standardized photographs of specimens. **c** Genotypes of loci most strongly associated with plumage differences inferred by admixture mapping on chromosomes 1A (43 loci) and around agouti signaling protein (*ASIP)* on chromosome 20 (49 loci) ordered by their position in the genome. Red and blue colors are homozygous genotypes typical for allopatric *alba* and *personata*, respectively, yellow are heterozygotes and white are missing data. **d** Heterozygosity plotted against hybrid index for genotypes shown on **c**. Dashed lines show limits in allopatric populations. **e** Putative inheritance mechanism of head plumage and frequency of genotypes found in each phenotypic group. P and p are *personata*-type alleles, A and a are *alba*-type alleles. Partially dominant alleles are capitalized. Asterisks on **c**–**e** indicate two *personata*-like hybrids with highly recombinant *ASIP* genotypes with a few *personata*-specific homozygous loci that may be key for determining the “masked” head plumage type.

To examine inheritance patterns of head plumage, we first assessed genotypic variation by extracting loci with the highest admixture mapping signal from the divergent regions on chromosome 1A (43 SNPs spanning ~138 kb) and on chromosome 20 (49 SNPs spanning 86 kb) (Fig. 3). The majority of SNPs in both regions were fixed to alternative states in allopatric populations, indicating that intermediate genotypes observed in the hybrid zone are due to hybridization and backcrossing (Fig. 3c). Within the hybrid zone, we used a hybrid index (a composite genotype scaled from 0 to 1) and heterozygosity at these loci to better characterize their contributions to hybrid phenotypes (Fig. 3d).

Parental genotypes were expected to have hybrid indices close to 0 (*alba*) or 1 (*personata*) and low heterozygosity. First-generation hybrid genotypes were expected to fall in the very top-center of each plot because they should have the highest heterozygosity and the most intermediate hybrid index possible, whereas more recombined genotypes of advanced-generation hybrids (i.e., F_2_ and backcrosses) should have lower heterozygosity and intermediate hybrid indices^27^. Backcrosses should have varying degrees of heterozygosity and hybrid indices closer to the respective parental genotypes^27^. To evaluate how distinct combinations of genotypic states match variation in head plumage, we categorized genotypes into “parental” states typical for allopatric populations and “intermediate” genotypes typical for hybrids and backcrosses.

### Inheritance of head plumage and its asymmetric introgression

Our findings suggest that the *ASIP* region has the major phenotypic effect, that there is partially dominant expression of *alba* alleles, and that the region interacts epistatically with the region on 1A. We originally inferred this architecture and mode of inheritance of *ASIP* (Fig. 3e) from the following observations. Carriers of mostly heterozygous *ASIP* genotypes (Ap) were found among *alba* and intermediate head plumage phenotypes, but most commonly in *alba*-like hybrids. Supporting the idea of dominant expression of *alba* phenotype, the majority of individuals with both an intermediate hybrid index and high heterozygosity for both genomic regions—likely F1 hybrids—had phenotypes of *alba*-like hybrids and less commonly had strict intermediate phenotypes (Fig. 3d). The “masked” plumage of *personata* and *personata*-like hybrids is typically produced when individuals possess a homozygous *personata*-type *ASIP* genotype (pp). Two *personata*-like hybrids (indicated by asterisks in Fig. 3c–e) exhibited recombinant *ASIP* genotypes but with at least two shared homozygous *personata* SNP genotypes that may be among those playing the primary role in head plumage coloration.

The phenotypic expression of the genomic region on chromosome 1A depends on the *ASIP* genotype. The *alba*-type *ASIP* homozygotes (AA) or heterozygotes (Ap) produce *alba* head plumage in combination with the *alba*-type 1A genotype (aa). These two *ASIP* genotypes produce *alba*-like hybrids or intermediate plumage types when combined with intermediate (aP) or *personata*-type (PP) genotypes on 1A. Most *personata*-like hybrids have *alba*-type (aa) or sometimes highly recombinant (aP) 1A genotypes combined with *personata*-type *ASIP* genotype (pp). The 1A heterozygotes (aP) typically have no phenotypic effect when combined with *personata*-type *ASIP* genotype (pp) and are commonly found in *personata* head phenotype. Interestingly, we did not observe any genotypes with *personata*-type genotype at 1A (PP) and the *alba*-type *ASIP* (AA), suggesting it is either a very rare direction of hybridization or that carriers of this genotype are inviable. To formally test the hypothesized mechanism of head plumage inheritance, we compared a series of models with additive/codominant, completely and partially dominant, and epistatic interactions. In these models, we followed an assumption of our working hypothesis that the regions on chromosomes 1A and 20 act as a two-locus bi-allelic system (although there are likely a few SNPs or haplotype blocks with key roles in influencing gene expression). The model with partially dominant inheritance of *personata* chromosome 1A alleles, partially dominant inheritance of *alba* chromosome 20 alleles and with epistatic interactions between the two loci best explained phenotypic variation (Supplementary Table 2). Among the determinants of model fit, interactions between chromosome 20 alleles had the largest effect (−54.2), followed by interactions between chromosome 1A alleles (−22.6) and the epistatic term (15.9).

**Fig. 4:**
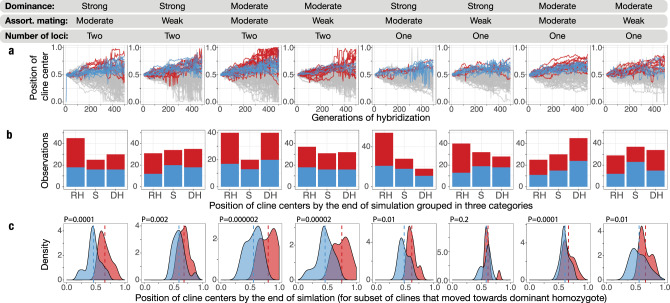
Simulations of temporal dynamics of hybridization where alleles for a reproductive barrier trait have partially dominant expression in one population, and with incomplete assortative mating. Throughout the figure, red and blue colors indicate mating trait and selectively neutral clines, respectively. **a** Position of cline centers (*Y* axis) over a course of 500 generations of hybridization (*X* axis). The eight scenarios vary in the strength of assortative mating, degree of partial dominance and a number of loci underlying mating trait. All clines have centers initially positioned at 0.5 and are allowed to move towards a trait encoded by recessive homozygote (0) or dominant homozygote (1). Clines moving towards dominant homozygote in mating trait and selectively neutral loci are highlighted. **b** Frequency of cline centers displaced towards recessive homozygote (RH, 0–0.44), staying relatively stable in space (S, 0.45–0.55) and displaced towards dominant homozygote (DH, 0.56–1) by the end of the simulation. **c** Distribution of cline center positions for selectively neutral and mating trait loci for a subset of mating clines moved towards dominant homozygote (DH). Mean centers are indicated by dashed lines. *P*-values stand for paired one-sided Wilcoxon test. Note that under the tested parameter combinations, the mating cline will rarely stay stable in space and will be moving towards recessive or dominant homozygote genotype. The latter scenario, matching the pattern observed in the wagtail hybrid zone, was an especially likely outcome under the moderate partial dominance and moderate assortative mating.

Given the partially dominant expression of the *alba* phenotype, we further explored whether this deviation from a codominant inheritance pattern could contribute to the observed asymmetric introgression of head plumage from *personata* into the *alba* genomic background. We used forward-time simulations^28^, where we assumed that the degree of dominance in an allele determining a phenotypic cue directly influences the probability of backcrossing (see the “Methods” section). We simulated geographic clines for one or two-locus mating traits, varying the dominance coefficient and the degree of assortativity. These simulations revealed that cline centers for mating trait loci will often not stay stable in space (Fig. 4). Interestingly, the direction of cline movement (i.e. towards dominant vs. recessive homozygote) was highly stochastic and variable both across and within the tested scenarios (Fig. 4a). In 30% (14–42%) of simulation outcomes, the cline moved towards the dominant homozygote, matching the scenario in the *alba* and *personata* hybrid zone (Fig. 4b). When the trait cline moved towards the dominant homozygote, in seven out of eight simulations the mating trait cline was significantly displaced from the neutral genomic background towards the dominant homozygote, again consistent with the observed patterns (Fig. 4c). The average magnitude of mating trait and genomic cline displacement predicated by our simulations (358.4 km across eight simulated scenarios, SD = 240.8) closely matched the observed displacement between the head plumage and ancestry clines in the wagtail hybrid zone (323 km^16^).

## Discussion

Identifying the molecular basis of traits targeted by divergent selection is a key step towards understanding the evolution of phenotypic variation. Here we show that a distinct plumage signal in a pair of white wagtail subspecies is associated with two narrow autosomal genomic regions, one of which contains the gene encoding *ASIP* and appears to have the major phenotypic effect. Further, our results suggest partially dominant expression of *ASIP* alleles: the mostly black head plumage of one subspecies (*personata*) is a homozygous recessive trait, while a significant proportion of heterozygotes exhibit the mostly white plumage of the other subspecies (*alba*). Our simulations suggest that this inheritance mechanism, in combination with plumage-mediated assortative mating, might contribute to asymmetric introgression of the *personata* plumage signal into the *alba* genomic background.

As we hypothesized, the simple segregation pattern of head plumage previously documented in the *personata*–*alba* hybrid zone corresponded to only two small genomic regions harboring a few genes. Importantly, our results suggest that non-coding genetic variation plays an important role in determining head plumage differences between wagtail subspecies, as has been found in other recent studies of plumage variation in birds^29–31^. Long runs of homozygosity upstream and downstream of *ASIP* in *personata*-type plumages likely overlap with promoters and enhancers of this gene. This finding adds to a growing collection of studies connecting genetic variation in *ASIP* and animal coloration^32–37^, and particularly highlights the role of regulatory genetic elements in evolutionary change^38–40^. Given that a few homozygous SNPs upstream and downstream of *ASIP* in highly recombinant phenotypes (Fig. 3c, d) are potentially sufficient to determine plumage differences (although we cannot rule out the role of structural genetic variation), it appears likely that these nucleotide substitutions play a role in changing the binding affinity of one or more transcription factors and regulation of *ASIP* expression. On chromosome 1A, a second major *F*_ST_ and admixture mapping peak (also located in a stretch of non-coding DNA) also appears to contribute to determination of head plumage, although with a minor phenotypic effect. The peak on 1A mostly occurs within non-coding sequence and the functional importance of this locus remains unclear. The nearby gene Parathyroid hormone-related protein (*PTHLH*) is involved in hair follicle development in mice^41^, suggesting its regulation might similarly affect feather patterning in birds. Supporting this hypothesis, *PTHLH* has been associated with plumage diversification in munias^33^. Moreover, two other genes (Fig. 2c) in the vicinity of the peak—phenylalanine hydroxylase (*PAH*) and tyrosinase hydroxylase (*TH*)—are involved in melanogenesis^42,43^. Finally, the region on 1A could be a trans-acting regulatory element affecting expression of *ASIP*—a possibility that is hard to evaluate using sequence data alone.

Our results suggest that mostly black *personata*-type plumage is largely determined by a recessive homozygous locus that includes *ASIP*. *ASIP* plays a critical role in pigment switching because it is an antagonist to the transmembrane receptor melanocortin 1 receptor (*MC1R*) that, when activated, promotes biochemical cascades of melanin production and darker coloration (reviewed in ref. ^36^). Expression of *ASIP* blocks *MC1R* signaling, resulting in reduced intensity of melanin pigmentation. The plausible explanation of head inheritance patterns observed in wagtails is that the regulatory effect of the *alba* allele increases expression of *ASIP* and thereby causes lighter coloration, and that a single gene dosage is sufficient to explain the partial dominance effect.

Given the strong positive assortative mating by head plumage and the fact that most *ASIP* heterozygotes resemble *alba* head phenotype, it appears likely that first-generation hybrids between *alba* and *personata* wagtails have a much higher likelihood of mating with *alba* than *personata*. This particularly opens up an opportunity for sex-biased introgression, if females choose mates based on head plumage, and F1 males are more likely to attract female *alba* than *personata*. Our simulations suggest that such a mechanism could contribute to the observed head plumage cline displacement under certain degrees of assortative mating and dominance of phenotypic alleles (Fig. 4), even in the absence of selective preference for *personata* phenotype. However, we emphasize that this outcome, while plausible, is a stochastic result of unpredictable hybridization dynamics under non-equilibrium inheritance of a trait involved in assortative mating, and it may differ when assortative mating is absent (e.g. ref. ^17^).

The genetic architecture of the plumage trait we documented in white wagtails (i.e., two epistatically interacting genomic regions) share properties with the genetic architecture of plumage color in the Carrion (*Corvus* (*corone*) *corone*) and Hooded (*Corvus* (*corone*) *cornix*) crows. In these taxa, plumage differences between darker and lighter melanin-based body coloration is governed by two genomic regions: one containing a major-effect gene norrin (*NDP*), the other harboring a cluster of genes with suppressed recombination that interact epistatically with *NDP*^29,44^. Moreover, simulations parameterized to crow plumage inheritance patterns suggest that assortative mating coupled with this epistatic genetic architecture can alone promote sexual trait introgression similar to results presented here^19^. Together, these results and our own findings support the long-standing hypothesis^17^, that peculiarities of barrier trait architecture governed by dominance, epistasis, or both, can drive asymmetric introgression. We stress, however, that the simulations alone do not rule out the possibility that the wagtail head plumage cline has moved as a consequence of other mechanisms. Although the displacement of the head plumage cline from the genomic background in wagtails was not associated with asymmetric mating preferences in social pairs^16^, selective advantage of one allele over another, social dominance, asymmetric aggressive behavior and skewed extra-pair mating all can promote asymmetric introgression^18,45,46^. Long-term monitoring of the *alba* and *personata* hybrid zone will be necessary to determine whether observed introgression patterns are due to whole zone movement (i.e. the ancestry cline is moving too, but at a slower rate) or asymmetric introgression of head plumage alone.

Substantial variation in head plumage and a complex evolutionary history in *Motacilla* wagtails have long puzzled biologists^20,21,47,48^. The reticulate nature of phenotypic variation in head and neck plumage^47^ suggests that variation in a small number of genes may underlie the rich phenotypic diversity in wagtails. Our study suggests that only two loci contribute to head plumage differences in *alba* and *personata* subspecies. Differentiation at the two loci is retained in allopatry despite hybridization and these loci, located on different chromosomes, introgress asymmetrically together from one population into another. These results suggest epistatic interactions contribute to the evolution of sexual signals and that the genetic architecture of a trait is an important determinant in introgression.

## Methods

### Sampling

We utilized 316 breeding individuals previously sampled along a 3000 km transect spanning the allopatric range of *alba* in western Siberia, the *alba–personata* hybrid zone in the western foothills of Altai mountains in Siberia, and the allopatric range of *personata* from southern Siberia to Uzbekistan^16^. Based on identifiable and discrete variation in head plumage (Supplementary Fig. 1), we selected 18 individuals with *alba* and 20 individuals with *personata* head plumage, 11 *alba*-like and nine *personata*-like hybrids, and four individuals that we refer to as “strictly intermediate”. All were males and originated from a ~100-kilometer zone of transition in the head plumage (Supplementary Table 1). In addition, we sequenced 10 males of each *alba* and *personata* from allopatric populations that are most remote from the areas of admixture: western Siberia (*alba*) and Uzbekistan (*personata*). A female *Motacilla samveasnae* collected in Stung Treng, Cambodia (Swedish Museum of Natural History 20016761/JWDKH13) and a male *M. citreola citreola* collected in Bayan Ovoo, Hentiy Aymag, Mongolia (University of Washington Burke Museum: 59798/CSW 5764) were used as outgroups.

### Whole genome sequencing and variant calling

Genomic DNA was extracted using DNeasy Blood & Tissue kit (Qiagen). Whole-genome libraries were prepared using Nextera XT (Illumina) v.2 kit, fragmented and 400–600 bp-long fragments were selected using BluePippin (Sage Science). Paired-end sequencing was performed on the NovaSEQ 6000 (Illumina) platform aiming to achieve ~10× coverage. Outgroup taxa were prepared separately using Illumina TruSeq PCR-free library preparations with a 350 bp insert size and sequenced on a NovaSEQ 6000 with a target sequencing coverage of 20×. We used FastQC v.0.11.7^49^ to assess read quality and Trimmomatic v.0.39^50^ to trim poor-quality reads and Illumina adapters with default settings. Next, bwa mem^51^ v.0.7.17-r1188 was used to align reads to the draft eastern yellow wagtail (*Motacilla tschutschensis tschutschensis*) reference genome (see below). Bam files were sorted, duplicates were marked, and files were indexed using samtools v. 1.3.1^52^ and picard-tools v.2.8.1^53^. Variants were called using HaplotypeCaller from GATK^54^ v.4.2. The resulting gvcfs were merged using CombineGVCFs and genotyped with GenotypeGVCFs followed by VariantFiltration using GATK-recommended filters. Finally, we used VCFtools^55^ v.0.1.15 to remove indels, keep only biallelic SNP with minor allele frequency above 5%, minQ > 20, min-meanDP > 4, max-meanDP < 75 and max-missing = 0.75. This resulted in a final dataset of 10,634,326 SNPs used for downstream analysis. Examples of all scripts are available via GitHub: https://github.com/erikrfunk/whole_genome_bioinformatics/blob/master/rosyfinch_notes.md. Raw read data associated with this project are publicly available as NCBI BioProject PRJNA690099 https://www.ncbi.nlm.nih.gov/bioproject/PRJNA690099.

### Assembling of the reference genome

The reference genome file was generated using ALLPATH-LG 4.7.0^56^ using a fragment library (180 bp) and two jumping libraries (3000 and 6000 bp) sequenced on an Illumina HiSeq 2500. We used MUMmer v.4.0 to establish the synteny between the yellow wagtail reference genome and a high-quality chromosome-level assembly of the zebra finch (*Taeniopygia guttata*) (https://www.ncbi.nlm.nih.gov/assembly/GCA_009859065.1/). We ran MUMmer for each zebra finch chromosome separately using default setting except increasing—maxgap to 1000 to boost from default of 90 to account for structural rearrangements in a divergent reference https://github.com/elinck/syma_speciation/blob/master/scripts/wgs_align_mummer.sh.

### Summary statistics, population structure, and windowed ABBA–BABA^57^

We used Python scripts developed by Simon Martin (“genomics_general”^26^) (https://github.com/simonhmartin/genomics_general) to estimate the relative *F*_ST_ (*K*_st_^58^ but with a modification to account for missing genotypes on a pair-by-pair basis) and the absolute *d*_*XY*_^59^ genomic divergence, and the nucleotide diversity^59^ in 25 kb sliding windows with 10 kb step. We used the default option (“coordinate”) to define the size of sliding windows. In this case, windows will cover a fixed range in the genome and if there are missing data, this can lead to variable numbers of sites used for each window. Per-site Weir-and-Cockerham *F*_ST_^60^ was estimated with VCFtools^55^. To test a hypothesis about introgression of *personata* alleles into *alba* genomic background, we utilized a further functionality of the “genomics_general” scripts to estimate windowed ABBA–BABA (*f*_d_)^26^. Here, we used a topology where P1 = sympatric *alba*, P2 = sympatric *personata*, P3 = allopatric *personata*, and P4 was designated as the outgroup. To estimate ancestral and derived allelic states, we polarized sites based on the genomes of a species closely related to the white wagtail (*M. samvesnae)* and a more distantly related member of the genus (*M. citreola*)^21^. Because using a small number of SNPs per window can generate stochastic errors that may cause *f*_d_ to have meaningless values even when *D* is positive, the “genomics_general” documentation suggests a minimum window size that allows at least 100 biallelic SNPs per window. To meet these criteria, we imposed a 50 kb sliding window with a 10 kb step. This resulted in an average of 505 loci per window (range: 176–1020). If shared variation between P3 and P2 (positive *D*) is of interest, then *f*_d_ might not be the best approach. Therefore, we utilized an alternative statistic *f*_dM_^26^, to better fit this scenario. Both metrics revealed similar patterns. We used principal component analysis (PCA) to assess population structure, and to summarize genomic variation of regions of interest. For the analysis of whole genome population structure, we thinned the dataset to retain one SNP per 10 kb window to avoid using loci in physical linkage. All SNPs were used for PCA in divergent regions on chromosomes 1A and 20. We ran PCA on the mean-centered genetic covariance matrix of SNP genotypes using the R v.3.6.1 (RStudio v.1.1.453) function *prcomp*.

### Gene annotation

We used MAKER v.2.31.10 pipeline (https://www.yandell-lab.org/software/maker.html) to annotate focal scaffolds in the yellow wagtail reference genome using zebra finch (http://useast.ensembl.org/Taeniopygia_guttata/Info/Index?redirect=no) protein and cDNA databases. Genes were predicted using SNAP (see MAKER documentation), which was trained iteratively until gene models converged. A few gene models had poor convergence between consecutive SNAP iterations. Therefore, as an alternative method we confirmed gene models within regions of divergence using the Flo lift over pipeline (https://github.com/wurmlab/flo) again using the Zebra Finch annotations (taeGut1: GCA_000151805.2).

### Quantification of plumage variation

Coloration of head and neck in white wagtails varies as a proportion of white unpigmented area on an otherwise black, melanin-based plumage. In *personata*, only a small “mask” is unpigmented, whereas *alba* has white ear coverts, malar area, and neck sides (Fig. 1c). Hybrids demonstrate a few categories of intermediate plumage (Supplementary Fig. 1). We used photographs of study skins taken in a standardized position, with a ruler grid and a white balance card. Images were pre-processed in Adobe Photoshop CC 2019 (Adobe) to standardize white balance. The total number of white pixels on the head and neck sides was estimated using ImageJ^61^ with standardized threshold settings (see ref. ^16^ for details).

### Admixture mapping

We used GEMMA v.0.98 (genome-wide efficient mixed-model association)^62^ to conduct admixture mapping for head plumage. To minimize the effects of isolation-by-distance and population structure we used only samples (*n* = 62) from the *alba* and *personata* hybrid zone. To estimate effects of isolated SNPs, we ran linear mixed-effects models (LMM, -lmm 1 option of GEMMA). To account for potential relatedness between individuals, we estimated a relatedness matrix using the -gk 1 option of GEMMA, which then was supplied as a covariate for LMM and BSLMM analyses used to assess genetic architecture of head plumage. For BSLMM, we ran four chains with a burn-in of five million steps and a subsequent 20 million MCMC steps sampling every 1000 iterations. We assessed four BSLMM hyperparameters: PVE (the proportion of variance explained by all SNPs), PGE (the proportion of genetic variance explained by alleles with measurable effect), posterior number of SNPs explaining trait variance in the model and gamma parameter assessing the proportion of variance explained by an individual locus of interest. We used a leave-one-out cross-validation approach to assess the posterior predictive power of BSLMM. More specifically, we excluded phenotypic information for one individual at a time and used the remaining dataset (*n* = 61) to predict its phenotype based on genotype by running BSLMM with the setting indicated above. Linear regression was then used to estimate the proportion of variance explained by predicted phenotypes as a measure of predictive performance^57^.

### Testing for dominance and epistasis

Given an apparent non-linear relationship between genotypic and head plumage variation, we compared a series of inheritance models with additive/codominant, partially dominant and completely dominant inheritance modes for genomic regions on chromosomes 1A and 20, and with epistatic interactions between 1A and 20. In these models, we encoded genotypes in the two genomic regions as *alba*-type, heterozygotes, or *personata*-type based on hybrid index and heterozygosity (Fig. 3d). We excluded three *personata*-like hybrids (1766, 1787, and 1842) due to highly recombinant genotypes and missing data for some loci that made it their genotype classification ambiguous. We then used linear models to assess power and contribution of each term in predicting head plumage values^63^.

### Geographic clines

We re-analyzed published data for the wagtail hybrid zone (https://search.datacite.org/works/10.5061/dryad.fg49r) to construct geographic cines for head plumage and ancestry. We summarized genomic variation using PCA and used PC1 scores as a proxy for ancestry^16^. Quantitative estimates for head plumage were taken as described above. We fit empirical data to five geographic cline models as described in ref. ^64^ using Metropolis–Hastings MCMC algorithm implemented *HZAR*^65^ v.0.2–5 package in R.

### Runs of homozygosity

We used the R package *detectRUNS*^66^ v.0.9.6 to search for homozygosity-rich regions in the two differentiated genomic regions on the chromosomes 1A and 20. We applied a window size of 12 loci, minSNP = 11 (minimum number of homozygous/heterozygous SNP in the window) and minLengthBps = 9000, with the rest of parameters being as described in the manual (https://cran.r-project.org/web/packages/detectRUNS/vignettes/detectRUNS.vignette.html).

### Hybrid zone simulations

To evaluate whether partial dominance of a plumage trait involved in mate choice might lead to displacement between phenotypic and genomic background clines, we ran forward-time simulations using a modified version of the R program *HZAM*^28^ v.2.0.0, which models one-dimensional hybrid zone dynamics in the presence of assortative mating, underdominance, and other processes. Our modification extended *HZAM*’s base features to support partial dominance of mating trait loci, using a user-defined dominance coefficient indicating the proportion of the homozygous dominant phenotype expressed by heterozygotes. Full code and an interactive web app implementing it can be found at https://github.com/elinck/hzam_shiny. Given that available evidence suggests that two regions on chromosomes 1A and 20 effectively work as a two-locus system (with the locus on chromosome 20 having the major effect) in determining head plumage in wagtails, we focused our simulations on genetic architectures with one or two mating trait loci. We ran 50 replicate simulations of 500 generations each with one or two mate choice loci and three neutral loci, 0.5 and 0.75 ratios of heterospecific to homospecific matings, a dominance coefficient of 0.5 and 0.75, a per-species carrying capacity of 800, initial range limits of 0–0.48 and 0.52–1 (given a total transect size of 1), a population growth rate of 1.05, a density dependence effect standard deviation of 0.01, and no loss of hybrid fitness. We fit clines for mating trait and neutral loci every five generations using a general additive model (GAM) implemented in the R package *mgcv*^67^ v.1.8–31 with the *gam()* function, using the formula *allele_frequency ~ s(transect_location)* and the "P-ML" method. We then used the *predict_gam()* function in the R package *tidymv* v.2.2.0 (https://github.com/stefanocoretta/tidymv) to generate model predictions for evenly spaced transect values, and used these to calculate cline width and cline center. To calculate cline width and center, we used Irwin’s^28^ "eightieth percentile" approach, which defines hybrid zone width as the geographic area between the locations at which the GAM cline fit crosses HI (hybrid index) = 0.1 and HI = 0.9, and center as the region within half a dispersal distance from the location at which the cline fit crosses HI = 0.5. Using these data, we performed Wilcoxon paired one-sided signed rank tests to determine whether cline center differed between neutral and mating trait loci. To compare the degree of asymmetry between introgression patterns in our simulations and the wagtail hybrid zone, we scaled displacement between simulated cline centers via means of dispersal distance^63^.

### Reporting summary

Further information on research design is available in the Nature Research Reporting Summary linked to this article.

## Supplementary information

Supplementary Information

Peer Review File

Reporting Summary

## Data Availability

Raw read sequencing data associated with this project and the reference genome generated as a part of this study are available as NCBI BioProject PRJNA690099). Genomic datasets are publicly available at Dryad 10.5061/dryad.dv41ns1wv. Zebra Finch protein and cDNA databases used int this study can be accessed at http://useast.ensembl.org/Taeniopygia_guttata/Info/Index?redirect=no. DNA samples are available from the authors upon request.

## Code availability

Previously published code used in this study: https://github.com/erikrfunk/whole_genome_bioinformatics/blob/master/rosyfinch_notes.md, https://github.com/elinck/syma_speciation/blob/master/scripts/wgs_align_mummer.sh, https://github.com/simonhmartin/genomics_general, https://github.com/wurmlab/flo. Custom code generated as a part of this study: https://github.com/elinck/wagtails [10.5281/zenodo.4321544], https://github.com/elinck/hzam_shiny [10.5281/zenodo.4321524], https://github.com/georgysemenov/wagtails [10.5281/zenodo.4319060].
