# Supplementary material for: Asymmetric introgression reveals the genetic architecture of a plumage trait
Source: Nat Commun. 2021 Feb 15;12:1019. doi: 10.1038/s41467-021-21340-y (PMC7884433; doi:10.1038/s41467-021-21340-y)
Supplement: Supplementary file 1 — Supplementary Information [file 41467_2021_21340_MOESM1_ESM.pdf]

Supplementary table 1. Sampling and phenotypic information. “Sample_ID” stands for the field-assigned specimen ID. Column “Amount of white on head and neck, th.pxls” contains information on the number of white pixels measured on standardized photographs of study skins and used for admixture mapping. “Phenotypic group” has been assigned based on categorical variation in head coloration (see figure 1 below) and was used for genomic scans for differentiation and screening for homozygosity runs. Samples and additional information can be obtained from the authors upon request.

Sample_ID	Geographic_group	Latitude	Longitude	Amount of white on head and neck, th.pxls	Phenotypic_group
DRK0381	Allopatric_alba	71.3783	56.0385	78.7	Allopatric_alba
DRK0382	Allopatric_alba	71.3783	56.0385	77.0	Allopatric_alba
DRK0383	Allopatric_alba	70.7246	56.2850	72.6	Allopatric_alba
DRK0384	Allopatric_alba	70.1105	56.1988	66.2	Allopatric_alba
DRK0385	Allopatric_alba	70.4401	56.3053	66.4	Allopatric_alba
DRK0386	Allopatric_alba	70.4401	56.3053	72.5	Allopatric_alba
DRK0388	Allopatric_alba	70.6139	56.4219	75.0	Allopatric_alba
DRK0389	Allopatric_alba	70.6139	56.4219	62.3	Allopatric_alba
DRK0391	Allopatric_alba	70.8635	56.2716	87.2	Allopatric_alba
DRK0392	Allopatric_alba	70.8635	56.2716	69.5	Allopatric_alba
SGA1665	Hybrid zone	53.2220	84.6790	63.4	Sympatric_alba
SGA1678	Hybrid zone	52.8890	84.7530	68.2	Sympatric_alba
SGA1680	Hybrid zone	52.8890	84.7530	66.8	Sympatric_alba
SGA1687	Hybrid zone	53.2180	84.5050	61.8	Sympatric_alba
SGA1690	Hybrid zone	53.2180	84.5050	67.4	Sympatric_alba
SGA1694	Hybrid zone	52.9390	84.5930	70.5	Sympatric_alba
SGA1695	Hybrid zone	52.9390	84.5930	76.4	Sympatric_alba
SGA1700	Hybrid zone	52.8490	84.4820	66.0	Sympatric_alba
SGA1703	Hybrid zone	52.8490	84.4820	73.4	Sympatric_alba
SGA1717	Hybrid zone	52.7950	84.9230	67.4	Sympatric_alba
SGA1724	Hybrid zone	52.8030	84.8430	73.6	Sympatric_alba
SGA1733	Hybrid zone	52.7310	84.9590	60.5	Sympatric_alba
SGA1736	Hybrid zone	52.7310	84.9590	64.0	Sympatric_alba
SGA1737	Hybrid zone	52.7310	84.9590	57.5	Sympatric_alba
SGA1739	Hybrid zone	52.7310	84.9590	65.7	Sympatric_alba
SGA1774	Hybrid zone	52.6530	85.1720	78.5	Sympatric_alba
SGA1786	Hybrid zone	52.3840	85.6730	78.0	Sympatric_alba
SGA1798	Hybrid zone	52.2770	85.4430	64.4	Sympatric_alba
SGA1668	Hybrid zone	53.1150	84.5470	39.5	Alba-like_hybrid
SGA1686	Hybrid zone	52.8690	84.5430	56.6	Alba-like_hybrid
SGA1720	Hybrid zone	52.7950	84.9230	50.2	Alba-like_hybrid
SGA1728	Hybrid zone	52.7420	84.8080	61.5	Alba-like_hybrid
SGA1759	Hybrid zone	52.7340	85.0160	58.3	Alba-like_hybrid
SGA1767	Hybrid zone	52.6030	85.0620	44.3	Alba-like_hybrid
SGA1769	Hybrid zone	52.6030	85.0620	49.8	Alba-like_hybrid
SGA1770	Hybrid zone	52.6030	85.0620	44.5	Alba-like_hybrid
SGA1801	Hybrid zone	52.2150	85.4720	41.4	Alba-like_hybrid
SGA1812	Hybrid zone	52.2150	85.4720	55.4	Alba-like_hybrid
SGA1817	Hybrid zone	52.4490	85.4200	54.8	Alba-like_hybrid
SGA1683	Hybrid zone	52.8860	84.6400	44.0	Intermediate
SGA1760	Hybrid zone	52.7340	85.0160	36.4	Intermediate
SGA1771	Hybrid zone	52.6030	85.0620	39.9	Intermediate
SGA1854	Hybrid zone	52.0400	86.0660	30.2	Intermediate
SGA1693	Hybrid zone	52.9390	84.5930	18.9	Personata-like_hybrid
SGA1748	Hybrid zone	52.6640	84.9300	14.8	Personata-like_hybrid
SGA1749	Hybrid zone	52.6640	84.9300	11.5	Personata-like_hybrid
SGA1766	Hybrid zone	52.6030	85.0620	17.7	Personata-like_hybrid
SGA1787	Hybrid zone	52.3840	85.6730	13.5	Personata-like_hybrid
SGA1792	Hybrid zone	52.3540	85.3370	10.7	Personata-like_hybrid
SGA1795	Hybrid zone	52.2770	85.4430	22.9	Personata-like_hybrid

SGA1829	Hybrid zone	52.5550	85.3390	15.5	Personata-like_hybrid
SGA1842	Hybrid zone	52.1780	85.9300	29.9	Personata-like_hybrid
SGA1738	Hybrid zone	52.7310	84.9590	9.3	Sympatric_personata
SGA1765	Hybrid zone	52.6030	85.0620	14.2	Sympatric_personata
SGA1768	Hybrid zone	52.6030	85.0620	12.9	Sympatric_personata
SGA1778	Hybrid zone	52.6530	85.1720	8.9	Sympatric_personata
SGA1781	Hybrid zone	52.6530	85.1720	8.5	Sympatric_personata
SGA1785	Hybrid zone	52.3840	85.6730	8.4	Sympatric_personata
SGA1788	Hybrid zone	52.3700	85.5350	6.9	Sympatric_personata
SGA1791	Hybrid zone	52.3700	85.5350	8.7	Sympatric_personata
SGA1793	Hybrid zone	52.3540	85.3370	6.5	Sympatric_personata
SGA1799	Hybrid zone	52.2770	85.4430	9.1	Sympatric_personata
SGA1800	Hybrid zone	52.2150	85.4720	12.7	Sympatric_personata
SGA1802	Hybrid zone	52.2150	85.4720	10.1	Sympatric_personata
SGA1803	Hybrid zone	52.2150	85.4720	7.8	Sympatric_personata
SGA1804	Hybrid zone	52.2150	85.4720	6.7	Sympatric_personata
SGA1807	Hybrid zone	52.2150	85.4720	8.6	Sympatric_personata
SGA1809	Hybrid zone	52.2150	85.4720	8.6	Sympatric_personata
SGA1818	Hybrid zone	52.4490	85.4200	13.6	Sympatric_personata
SGA1832	Hybrid zone	52.5550	85.3390	13.6	Sympatric_personata
SGA1833	Hybrid zone	52.5550	85.3390	13.4	Sympatric_personata
SGA1838	Hybrid zone	52.1720	85.8560	11.6	Sympatric_personata
SGA1536	Allopatric_personata	41.3100	69.4400	14.6	Allopatric_personata
SGA1539	Allopatric_personata	41.3100	69.4400	11.0	Allopatric_personata
SGA1541	Allopatric_personata	41.3100	69.4400	14.3	Allopatric_personata
SGA1542	Allopatric_personata	41.3100	69.4400	12.9	Allopatric_personata
SGA1545	Allopatric_personata	41.3100	69.4400	13.1	Allopatric_personata
SGA1546	Allopatric_personata	41.3100	69.4400	9.2	Allopatric_personata
SGA1548	Allopatric_personata	40.9100	69.7900	9.2	Allopatric_personata
SGA1550	Allopatric_personata	40.9500	69.8300	10.4	Allopatric_personata
SGA1554	Allopatric_personata	40.9500	69.8300	7.8	Allopatric_personata
SGA1561	Allopatric_personata	40.9500	69.8300	14.9	Allopatric_personata

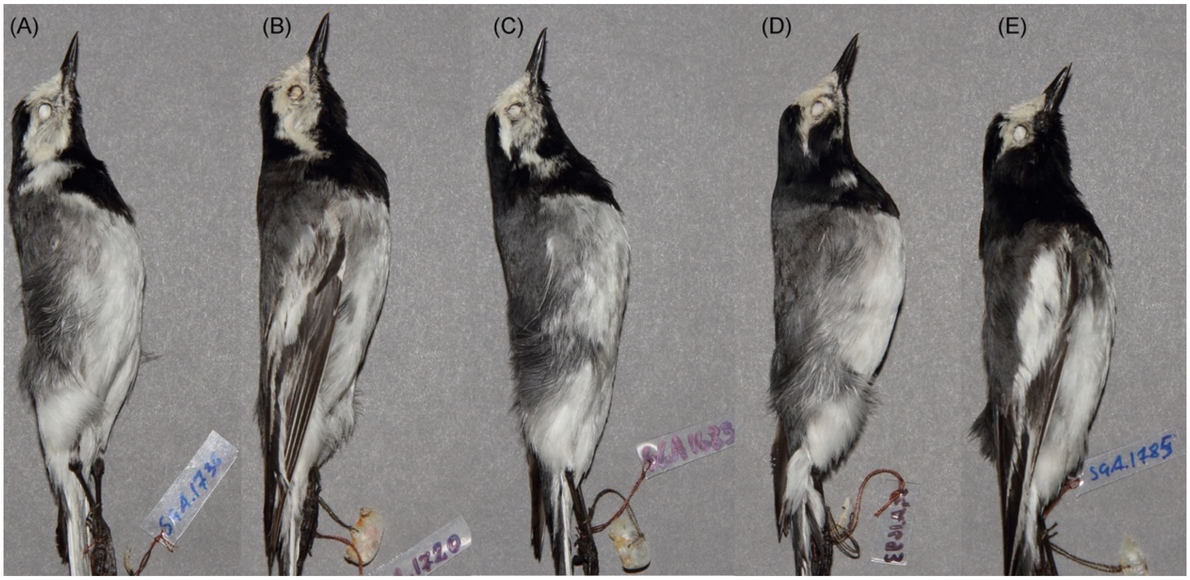

Supplementary figure 1. Variation in head and neck plumage observed in the *alba* and *personata* hybrid zone: (A) Parental *alba*, (B) *Alba-like* hybrid, (C) Intermediate, (D) *Personata*-like hybrid, (E) Parental *personata.* Tags are sample IDs (table 1 above).

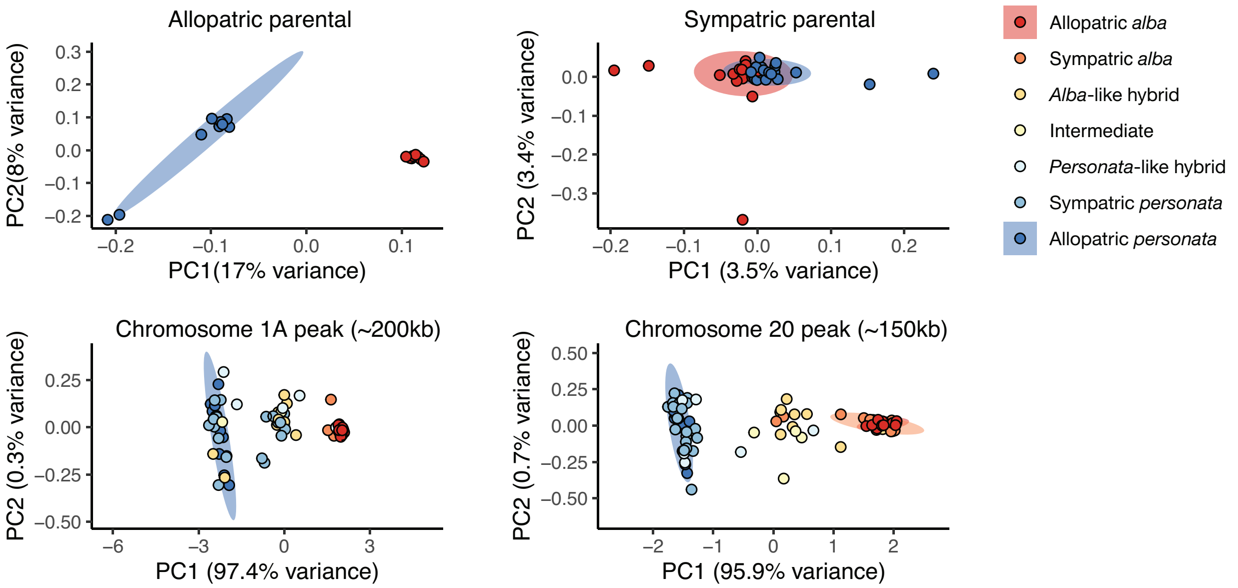

Supplementary figure 2. Principal Component Analysis (PCA) of genomic variation in *alba* and *personata*. For the whole-genome PCA (top) we thinned our SNP dataset to avoid loci in tight physical linkage (1 SNP per 10,000bp window kept), resulting in 106,560 and 104,427 loci for allopatric and sympatric comparisons respectively. Note that while allopatric populations are clearly separated along PC1 suggesting population structure, it is not the case in sympatric *alba* and *personata*. Bottom plots show PCAs for two genomic regions differentiating sympatric *alba* and *personata* on chromosomes 1A (3,080 loci) and 20 (1,605 loci). Note much better correspondence between plumage classification and variation in the chromosome 20 peak, compared to 1A. Ellipses are 95% CI for allopatric populations.

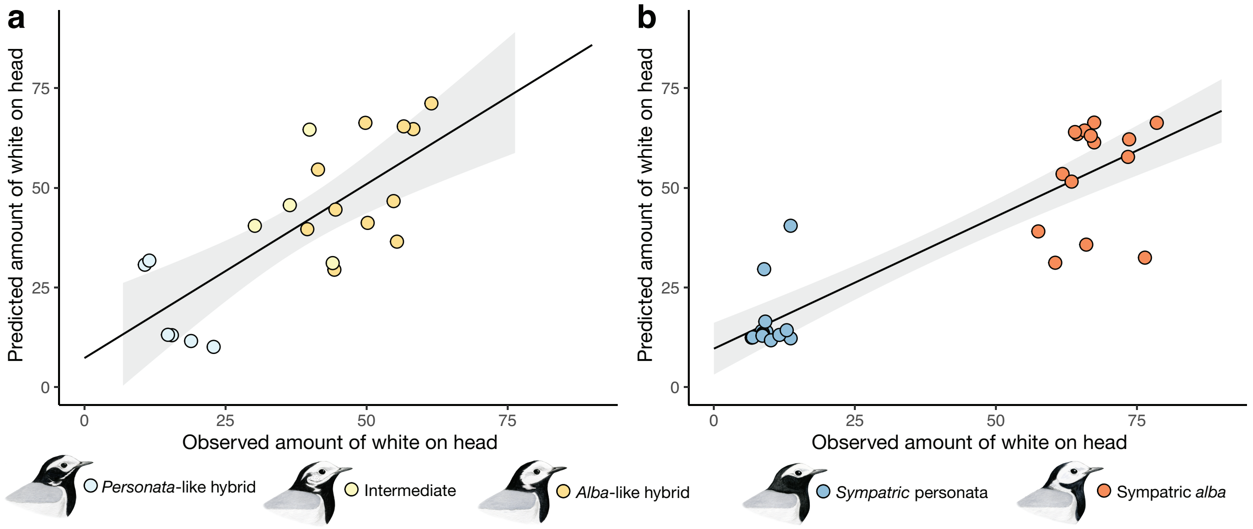

Supplementary figure 3. Results of leave-one-out cross validation of the predictive power of the Bayesian Sparse Linear Mixed Model of GEMMA. We consecutively excluded phenotypic information for one individual and used the remaining dataset to predict its phenotypic value based on genotype. The predictive power was moderate for hybrids (a, adjusted R^2 = 0.54) and higher for sympatric parental phenotypes (b, adjusted R^2 = 0.78), consistent with non-linear relationships between allelic and plumage variation. Shading shows the 95% CI for the regression line.

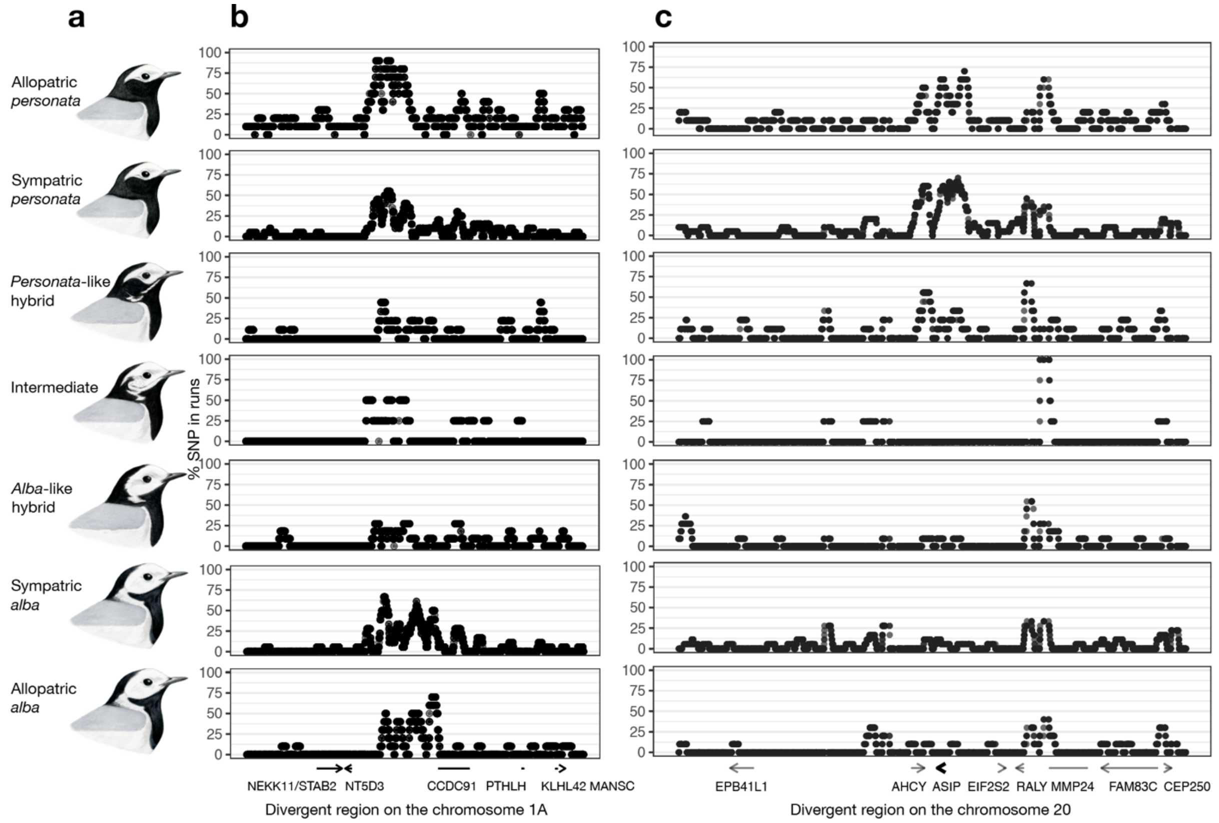

Supplementary figure 4. Divergent regions on chromosomes 1A and 20 contain long runs of homozygous genotypes in some phenotypic groups. a, Typical phenotypes for each group. b and c, Runs of homozygosity for each phenotypic group in and around of the divergent regions on the chromosomes 1A and 20. Note that sample size varies among phenotypic groups resulting in a variable step size on Y axis, that is particularly large in the "Intermediates" (n=4). Allopatric and sympatric *alba* and *personata* have long stretches of homozygosity on chromosome 1A, which are less prominent in intermediate plumage types (*alba*-like hybrids, intermediates, *personata*-like hybrids), likely due to phenotypic expression of the region on 1A being substantially dependent on *ASIP* genotype from chromosome 20. Note that "masked" phenotypes (*personata* and *personata*-like hybrids) are highly homozygous for the region around *ASIP*. This is not the case in other phenotypic groups, suggesting that *alba*-type phenotypes are expressed in intermediate genotypes, supporting the idea of partially dominant expression of *alba* ASIP alleles.

Supplementary table 2. Comparison of inheritance models for head plumage with additive/codominant, complete and partially dominant allelic interactions, and with epistatic effects. In these models we made an assumption that head plumage is effectively encoded as two-locus system. We classified genotypes from the genomic regions on chromosomes 1A and 20 as *alba*-type, heterozygote or *personata*-type based on observed hybrid index and heterozygosity. We then used linear models to estimate the predictive power of different inheritance models on plumage variation. Additive/codominant inheritance implies that heterozygote genotypes are strictly intermediate, and phenotypes are a simple sum of the effects of two loci. In the partially dominant model, heterozygotes have phenotypes 25% more similar to the respective dominant homozygote. In the complete dominance models, heterozygotes and dominant homozygotes have identical phenotypes. The epistatic term stands for interactions between 1A and 20 genotypes. The best model is boldfaced.

Model	df	AIC	AICc	BIC
Additive/codominant inheritance of 1A and 20 alleles	4	442.3	443.1	450.7
Additive inheritance of 1A and 20 alleles + epistasis	5	444.3	445.5	454.7
Partial dominance of *personata* 1A and *alba* 20 alleles	4	427.4	428.1	435.7
**Partial dominance of *personata* 1A and *alba* 20 alleles + epistasis**	5	423.3	424.4	433.7
Partial dominance of *alba* 1A and *personata* 20 alleles	4	478.8	479.5	487.1
Partial dominance of *alba* 1A and *personata* 20 alleles + epistasis	5	477.9	479.0	488.3
Partial dominance of *alba* 1A and *alba* 20 alleles	4	441.5	442.2	449.8
Partial dominance of *alba* 1A and *alba* 20 alleles + epistasis	5	434.6	435.7	445.0
Partial dominance of *personata* 1A and *personata* 20 alleles	4	475.7	476.5	484.0
Partial dominance of *personata* 1A and *personata* 20 alleles + epistasis	5	475.0	476.1	485.4
Complete dominance of *personata* 1A and *alba* 20 alleles	4	441.4	442.2	449.7
Complete dominance of *personata* 1A and *alba* 20 alleles + epistasis	5	430.8	431.9	441.2
Complete dominance of *alba* 1A and *personata* 20 alleles	4	509.2	509.9	517.5
Complete dominance of *alba* 1A and *personata* 20 alleles + epistasis	4	509.2	509.9	517.5

Complete dominance of *alba* 1A and *alba* 20 alleles	4	470.0	470.7	478.3
Complete dominance of *alba* 1A and *alba* 20 alleles + epistasis	5	464.5	465.6	474.9
Complete dominance of *personata* 1A and *personata* 20 alleles	4	503.7	504.4	512.0
Complete dominance of *personata* 1A and *personata* 20 alleles + epistasis	5	505.3	506.4	515.7
