# Supplementary material for: Asymmetric introgression reveals the genetic architecture of a plumage trait
Source: Nat Commun. 2021 Feb 15;12:1019. doi: 10.1038/s41467-021-21340-y (PMC7884433; doi:10.1038/s41467-021-21340-y)
Supplement: Supplementary file 3 — Reporting Summary [file 41467_2021_21340_MOESM3_ESM.pdf]

nature research

Corresponding author(s): Georgy Semenov

Last updated by author(s): 07/02/2020

Reporting Summary

Nature Research wishes to improve the reproducibility of the work that we publish. This form provides structure for consistency and transparency in reporting. For further information on Nature Research policies, see our Editorial Policies and the Editorial Policy Checklist.

Statistics

For all statistical analyses, confirm that the following items are present in the figure legend, table legend, main text, or Methods section.

n/a	Confirmed	
☐	☒	The exact sample size (*n*) for each experimental group/condition, given as a discrete number and unit of measurement
☐	☒	A statement on whether measurements were taken from distinct samples or whether the same sample was measured repeatedly
☐	☒	The statistical test(s) used AND whether they are one- or two-sided<br>*Only common tests should be described solely by name; describe more complex techniques in the Methods section.*
☐	☒	A description of all covariates tested
☐	☒	A description of any assumptions or corrections, such as tests of normality and adjustment for multiple comparisons
☐	☒	A full description of the statistical parameters including central tendency (e.g. means) or other basic estimates (e.g. regression coefficient) AND variation (e.g. standard deviation) or associated estimates of uncertainty (e.g. confidence intervals)
☐	☒	For null hypothesis testing, the test statistic (e.g. *F*, *t*, *r*) with confidence intervals, effect sizes, degrees of freedom and *P* value noted<br>*Give P values as exact values whenever suitable.*
☐	☒	For Bayesian analysis, information on the choice of priors and Markov chain Monte Carlo settings
☐	☒	For hierarchical and complex designs, identification of the appropriate level for tests and full reporting of outcomes
☐	☒	Estimates of effect sizes (e.g. Cohen's *d*, Pearson's *r*), indicating how they were calculated

Our web collection on statistics for biologists contains articles on many of the points above.

Software and code

Policy information about availability of computer code

Data collection	Adobe Photoshop 2020 v. 21.20, ImageJ 1.46r
Data analysis	FastQC v.0.11.7, Trimmomatic v.0.39, bwa mem v.0.7.17-r1188, samtools v. 1.3.1, picard-tools v.2.8.1, GATK v.4.2, VCFtools v.0.1.15, ALLPATH-LG 4.7.0 MUMmer v.4.0, R v.3.6.1 (packages HZAR v.0.2-5, detectRUNS v.0.9.6, HZAM v.2.0.0, mgcv v.1.8-31, tidymv v.2.2.0, R-core function prcomp), RStudio v.1.1.453, MAKER v.2.31.10, GEMMA v.0.98. Previously published code: https://github.com/erikrfunk/whole_genome_bioinformatics/blob/master/rosyfinch_notes.md, https://github.com/elinck/syma_speciation/blob/master/scripts/wgs_align_mummer.sh, https://github.com/simonhmartin/genomics_general, https://github.com/wurmlab/flo. Custom code generated as a part of this study: https://github.com/elinck/wagtails [DOI https://doi.org/10.5281/zenodo.4321544], https://github.com/elinck/hzam_shiny [DOI https://doi.org/10.5281/zenodo.4321524], https://github.com/georgysemenov/wagtails [DOI https://doi.org/10.5281/zenodo.4319060]. Zebra Finch protein and cDNA database http://useast.ensembl.org/Taeniopygia_guttata/Info/Index?redirect=no. DNA samples are available from the authors upon request.

For manuscripts utilizing custom algorithms or software that are central to the research but not yet described in published literature, software must be made available to editors and reviewers. We strongly encourage code deposition in a community repository (e.g. GitHub). See the Nature Research guidelines for submitting code & software for further information.

Data

Policy information about availability of data

All manuscripts must include a data availability statement. This statement should provide the following information, where applicable:

- Accession codes, unique identifiers, or web links for publicly available datasets
- A list of figures that have associated raw data
- A description of any restrictions on data availability

Raw read and reference genome data associated with this project are publicly available at (NCBI BioProject PRJNA690099). Datasets used for genomic analyses are available at DRYAD DOI https://doi.org/10.5061/dryad.dv41ns1wv.

Field-specific reporting

Please select the one below that is the best fit for your research. If you are not sure, read the appropriate sections before making your selection.

☐ Life sciences ☐ Behavioural & social sciences ☒ Ecological, evolutionary & environmental sciences

For a reference copy of the document with all sections, see nature.com/documents/nr-reporting-summary-flat.pdf

Ecological, evolutionary & environmental sciences study design

All studies must disclose on these points even when the disclosure is negative.

Study description	Study uses whole-genome sequencing data to dissect genetic architecture and inheritance patterns of a divergent mating signals in two hybridizing bird subspecies (Motacilla alba alba and M. a. personata) via admixture mapping. Forward time simulations were used to test if genetic architecture of the mating signal can explain its observed asymmetric introgression patterns.
Research sample	Specimens were sampled across wild populations in the alba and personata hybrid zone in Siberia and from parental allopatric populations away from the hybrid zone. While comparison between allopatric samples was used to infer the "basal" level of genomic differentiation between parental lineages, samples from the hybrid zone were used to identify genomic regions resisting admixture. The dataset used for admixture mapping was sampled only within the hybrid zone to minimize the effects of population structure. The dataset was designed to capture common alleles in genomic scans of differentiation (n=10 of each population). For admixture mapping, we aimed to include equal representation of parental (n=20) and each of intermediate phenotypic groups (n=10) whenever possible, again to capture common genetic variants.
Sampling strategy	Dataset used in this study is a subset from a larger pool of 316 individuals sampled on a 3,000 kilometer transect across alba and personata hybrid zone (Semenov et al. 2017). Only males were used for analysis to avoid allele frequency bias for Z chromosome. Sample sizes for allopatric population were determined to be representative of population allele frequencies (n=10). Since we were interested in a balanced representation of plumage categories for admixture mapping, we used plumage phenotype in the selection process. The priority was given to study skins with the best preparation quality. Sampling for the hybrid zone aimed to represent parental phenotypes in approximately equal proportions (n=18 of parental alba and n=20 of parental personata) and be sufficient to capture rare genetic variants. Intermediate plumage categories were taken with approximately equal proportions (11 alba-like hybrids and 9 personata-like hybrids), except for intermediate plumage type which were extremely rare across the hybrid zone (we were able to find only four among ~200 screened males).
Data collection	Specimens were collected and field-preserved as study skins with a sample of breast muscle for DNA extraction preserved in 100% ethanol. All study skins were prepared by a single person (GAS) to minimize the effect of different taxidermists on study skin size and proportions. Data recording (field journal) in the field was performed by a single person (DRK) and checked by GAS to minimize potential for errors. Phenotypic data were collected using standardized digital photographs taken using Canon 60D camera on a neutral gray background and a white/back reference card.
Timing and spatial scale	Sampling took place during the breeding season to ensure only local breeding individuals are collected (and hence no migrants with unknown origin had a potential to bias our population genetic inferences) - allopatric personata in Uzbekistan: May 1- May 7, 2012; Hybrid zone in Siberia: June 1 -June 20, 2012; allopatric alba in west Siberia: June 13 -June 15, 2014. Optimal windows for sampling were determined using literature on wagtail life history. Breeding status of individuals was further confirmed by gonad examination. Specimens were collected from a 3,000 kilometer transect with the major sampling effort made in a ~100 kilometer-wide hybrid zone (approximate limits of the hybrid zone and areas where minimal/no introgression from the hybrid zone was expected were known from previously published data, museum collections and our own research).
Data exclusions	Sequencing data from three individual were excluded due to sequencing depth of coverage being a few times lower than the rest of the dataset (decision to exclude these individuals was made after examining sequence depth in the complete dataset).
Reproducibility	We took three replicates of phenotypic measurements to ensure consistency. All replicated attempts were consistent and successful.
Randomization	Samples were assigned into phenotypic groups based on well-marked and well-identifiable phenotypic differences (See supplementary figure 1) between parental populations, and between parental phenotypes in the hybrid zone and phenotypic hybrids. This group assignment was further controlled by assessing quantitative plumage estimates for each individual. For admixture mapping, we only used quantitative phenotypic estimates without group information. For the scans of genomic differentiation between parental sympatric phenotypes we chose individuals with plumage measurements within variation limits of allopatric populations.

Blinding	Blinding was not relevant to our study design. For genomic comparisons we used geographically separated populations or distinct, well-identifiable phenotypes. For admixture mapping we did not assign specimens to groups.

Did the study involve field work? [x] Yes [ ] No

Field work, collection and transport

Field conditions	Sampling took place in the primary wagtail habitat such as near farms, mills, banks of rivers and lakes. Temperatures were mostly above freezing during the day time with occasional cold snaps in the night. We did not register rainfall during our fieldwork.
Location	Allopatric personata - Uzbekistan (near towns Chirchik, Nurobad, Akchasai). Hybrid zone - Siberia (between Gorno-Altaisk and Barnaul towns). Allopatric alba - west Siberia (near towns Repyeva, Bystrukha, Kareglazova, Konevo, Panovskoe, Repyeva, Yaman). For GPS coordinates of sampling locations please see Supplementary table 1 in supplementary materials asscoiated with this paper.
Access & import/export	All specimens were collected with necessary permits (issued by Departament Po Okhrane Zhivotnogo Mira Novosibirskoy Oblasti in 2014, Glavnoye Upravleniye Prirodnykh Resursov I Ekologii Altayskogo Kraya in 2012 and Upravleniye Rossel'khoznadzora Po Altayskomu Krayu I Respublike Altay in 2012) granted to the Institute of Systematics and Ecology of Animals, Novosibirsk, Russia. Extracted DNA (which import is not formally regulated) was imported to the U.S.
Disturbance	The white wagtail is an abundant species with the least concern conservation status. In each sampling locality only a small fraction of breeding individuals was collected (<< 1%). Disturbance was minimized by conducting fieldwork in as timely manner as possible.

Reporting for specific materials, systems and methods

We require information from authors about some types of materials, experimental systems and methods used in many studies. Here, indicate whether each material, system or method listed is relevant to your study. If you are not sure if a list item applies to your research, read the appropriate section before selecting a response.

Materials & experimental systems

n/a	Involved in the study
[x]	[ ] Antibodies
[x]	[ ] Eukaryotic cell lines
[x]	[ ] Palaeontology and archaeology
[ ]	[x] Animals and other organisms
[x]	[ ] Human research participants
[x]	[ ] Clinical data
[x]	[ ] Dual use research of concern

Methods

n/a	Involved in the study
[x]	[ ] ChIP-seq
[x]	[ ] Flow cytometry
[x]	[ ] MRI-based neuroimaging

Animals and other organisms

Policy information about studies involving animals; ARRIVE guidelines recommended for reporting animal research

Laboratory animals	Study did not include experiments with laboratory animals
Wild animals	Two subspecies of the white wagtail Motacilla alba alba and M. a. personata were collected. Only males were used in this study to avoid potential noise in data introduced by sexual dimorphism (also see note on Z chromosome allele frequencies above). Specimens were euthanized in order to preserve phenotypic information in a form of study skins for this and future studies and allow gonad examination to ensure local breeding status.
Field-collected samples	Specimens were immediately euthanized upon capture. Collected specimens were processed on the same or next day to ensure high quality of skins and tissue material for DNA extraction. Study skins are publicly available at the Siberian Zoological Museum at the Institute of Systematics and Ecology of Animals in Novosibirsk (ISEA), Russia (field IDs DRK0381-0392; SGA1536-1838; museum IDs 8322-8629). Tissue specimens are available at ISEA under the same field IDs.
Ethics oversight	Sampling was approved by the Institute of Systematics and Ecology of Animals program of biodiversity collections (Bird Ecology Research Group committee led by Alexander Yurlov).

Note that full information on the approval of the study protocol must also be provided in the manuscript.
