# Supplementary material for: Asymmetric introgression reveals the genetic architecture of a plumage trait
Source: Nat Commun. 2021 Feb 15;12:1019. doi: 10.1038/s41467-021-21340-y (PMC7884433; doi:10.1038/s41467-021-21340-y)
Supplement: Supplementary file 2 — Peer Review File [file 41467_2021_21340_MOESM2_ESM.pdf]

Reviewer #1 (Remarks to the Author):

Semenov et al analyze patterns of genomic variation/differentiation and genotype-phenotype associations across a wagtail hybrid zone. The key findings of the paper are that genetic differentiation between sympatric wagtail taxa is mostly limited to two regions, one on chromosome 1A and one on chromosome 20, that these regions are associated with a plumage phenotype in hybrids, and that one of the regions contains the gene agouti (which is associated with variation in melanic phenotypes in many species). They further show that genotype-phenotype associations at these loci are consistent with dominance and epistasis, which could explain asymmetric introgression of color phenotypes.

I think the paper is generally clear and places the work in appropriate context. The work is based on appropriate data and sound methods (the sample sizes are a tad small, but fine given the genetic architecture of the trait). I found the interpretation of the results reasonable. As noted by the authors, there is increasing evidence in several systems that dominance and epistasis shape patterns of genetic and phenotypic variation in nature. This work nicely adds to the story and does so in a system with a tractable genetic architecture.

My main suggestions and criticisms concern the need (or benefit) of additional formal analyses to strengthen and make more quantitative some of the key results. In particular:

1. I agree that the genotype-phenotype associations presented are consistent with dominance and epistasis, but this is never formally assessed nor are coefficients of dominance and epistasis estimated. I think additional analyses to formally quantify dominance and epistasis would really strengthen the paper. I realize that including dominance and especially all of the pairwise epistasis terms for all SNPs in the BSLMM would be intractable. But there are other options that should work. (i) You could treat (code) each of the two associated regions as a bi-allelic locus with alba and personata alleles and then fit a linear model with additive, dominance and epistasic terms to estimate the magnitude of each of these effects. Based on Figure 3, this would mostly work, though there would be some ambiguity in the encoding for a few individuals. (ii) You could do something like (i) but with each region split into ancestry blocks that show no (very little) evidence of recombination in the data set (to get around ambiguity in coding at the region/locus level). (iii) You could fit a model akin to (i) by sampling SNPs from the associated regions on 1A and 20 based on their posterior inclusion probabilities from the BSLMM (with multiple samples from the posterior you could integrate over the genotype-phenotype association posterior). (iv) You could test for marginal epistasis for each SNP in the region on 1A vs all SNPs in the region on 20 and vice a versa (you can do this with MAPIT). (v) You could re-run the BSLMM in gemma with dominance for SNPs in just the regions on 1A and 20 associated with color and for epistasis only between the SNPs with evidence of marginal epistasis. You can then ask how this affects PVE.

2. I like the BSLMM approach, but think it is always good to assess the posterior predictive power of the model. In particular, I recommend repeating the analysis with leave-one-out cross validation to predict the phenotypes of each hybrid individual (one at a time) based on a model fit from all other individuals. One can then calculate the percent of the variation in observed phenotypes explained by the predicted phenotypes as a measure of predictive performance (this is especially useful in the context of comparisons of models with versus without dominance or epistasis; see (v) above).

3. The simulations add nicely to the paper, and the high variance in the outcomes across simulations is rather interesting. However, while the simulations show dominance and assortative mating can give rise to asymmetric introgression, they do not currently show that the magnitude (degree) of asymmetry is of the order seen in this data set. As these are meant (I think) to (roughly) approximate the wagtail hybrid zone, it would be nice to compare (with appropriate scaling) the magnitude of the effects seen in the simulations to what is seen in the wagtail hybrid zone. Also, given that the estimate for the number of causal variants was 5.3+-2.6 (posterior SD?), it might be useful to consider simulations with two genetic regions but multiple causal variants in each. Or at least to make the

choice of two loci for the simulations very clear.

4. I am always a bit skeptical of historical demographic inference. I would like to see that the model estimated does a good job of predicting/generating the observed data. In other words, the best parameter estimates given the general structure of the model might or might not provide an actual good fit for the data. Simulating/predicting data from the fit model can test this. Also, how confident are you in the mutation rate used? Some indication of this and the sensitivity to the mutation rate would be nice (this always worries me as being off by an order of magnitude seems possible in many cases and would shift dates by an order of magnitude).

Other minor comments and suggestions (with line numbers):

L25, reword to clarify whether "local" is in geographic space or along a chromosome (I assume the latter, but not totally clear)

L27-30, this assumes minimal coupling/the lack of a strong barrier to gene flow.

L61, clarify the distinction between abrupt and clinal (e.g., sharp versus gradual cline).

L68-69, provide the null expectation for mating between individuals of similar phenotypes based on their proportions to put 50% in context.

L135, fd is an admixture proportion, just estimated in the context of ABBA-BABA.

L177, you could be a bit more specific about these expectations, see, e.g., Fig. 5 from https://doi.org/10.1111/eva.12380 (consider adding the triangle lines to the figure, you can almost see the points making the lines as it is).

L194, here and elsewhere you refer to 'alba' alleles, but it is never totally clear whether you are essentially thinking of alleles at SNPs or two loci with haplotype blocks or something else. I realize this is a bit complicated, but being more specific in how you are using and thinking about this (and that you don't really know how many causal variants there are) would be nice.

L220, what is non-equilibrium inheritance?

L302, The PCA clusters, especially on 1A really suggest a SV or other mechanism for reduced recombination.

L428, I was a bit surprised to see such a high minor allele frequency (0.05) filter. I doubt it matters much for the paper, but is there a reason for it. Also, after filtering were genotypes called or was uncertainty in genotype taken into account?

L450, how were N's dealt with in reference, and in general unevenness in coverage across windows when calculating pi and dxy (as they divide by the number of bps in a window, which isn't really the same as the window size unless you have high coverage data across the whole window)

L507, not really large effects, just measurable (non-infinitesimal)

Zach Gompert

Reviewer #2 (Remarks to the Author):

In “Recent hybridization and asymmetric introgression reveal the genetic architecture of a plumage

trait," Semenov et al. investigate mechanisms underlying the geographic displacement of a pigmentation cline from clinal variation in genetic markers sampled from the rest of the genome in a pair of hybridizing bird subspecies. This displacement is odd because the pigmentation trait contributes to assortative mating between the subspecies, which should give rise to concordant clines. To explain this seemingly counterintuitive pattern (one that is seen in other hybrid zones as well), they sequenced whole genomes of phenotypically diverse individuals sampled from the hybrid zone plus allopatric parental populations. With these data, they examined patterns of genomic differentiation and genotype-phenotype association and found two regions that had both the highest levels of differentiation and strongest association with plumage phenotypes. Based on these analyses and on a BSLMM analysis that enabled them to estimate parameters related to genetic architecture (e.g., PVE and # of loci with "sparse" effects), they concluded that pigmentation differences were attributable to a simple genetic architecture. Next, they examined ancestry in these two regions in hybrids that fell into different phenotypic categories (e.g., parental-like, intermediate, etc.) to make inferences about dominance and epistasis. Using the inferred inheritance model, they simulated pigmentation and genomic clines under a variety of conditions to test the hypothesis that observed trait inheritance could explain the displacement of clines. Although cline movement appeared to be highly stochastic across simulations, they did find that when pigmentation clines were displaced, they had a strong tendency to be displaced towards the dominant homozygote, which is consistent with the observed cline. Finally, to put this cline in the proper historical context, they estimated demographic parameters under a model of secondary contact and found that the timing of hybridization coincides with anthropogenic changes to the habitat. Overall, these results contribute to our understanding of how trait genetic architecture can give rise to displaced clines, even for traits that mediate assortative mating. They also provide a likely empirical example of human-mediated hybridization.

This paper was an absolute pleasure to read. It is very well written, interesting, and addresses an important conundrum in evolutionary biology. There is a substantial amount of data included in the paper, the analyses are appropriate and reflect current best practices (to the best of my knowledge), the results are clearly communicated and interpreted, and the major conclusion of the paper--that the displacement of the pigmentation cline can be explained by trait genetic architecture--is supported by the data. The authors are also clear about the limitations of the study, acknowledging that cline movement is highly stochastic and other mechanisms could potentially explain the observed clines. I also like that the authors did not bog down the paper with a bunch of extraneous analyses and results that were unrelated to the central questions of the paper. I rarely encounter a paper that I think can be published as is, but I think this is one of those rare exceptions. And I am confident that these results would be of broad interest to readers of Nature Communications. Thanks to the authors for making my job as a reviewer so easy!

I had one minor comment:

L67-69: "Strong positive assortative mating by head plumage (~50% of pairs are formed between the same parental phenotypes)…" I read this line several times and am still confused. If 50% of pairs are same-phenotype, doesn't that mean that 50% of pairs are different-phenotype? That seems like random mating. Did the authors mean something else here? Maybe same-phenotype pairs are 50% more common? Please reword for clarity or explain how this is strong assortative mating.

Reviewer #1

Semenov et al analyze patterns of genomic variation/differentiation and genotype-phenotype associations across a wagtail hybrid zone. The key findings of the paper are that genetic differentiation between sympatric wagtail taxa is mostly limited to two regions, one on chromosome 1A and one on chromosome 20, that these regions are associated with a plumage phenotype in hybrids, and that one of the regions contains the gene agouti (which is associated with variation in melanic phenotypes in many species). They further show that genotype-phenotype associations at these loci are consistent with dominance and epistasis, which could explain asymmetric introgression of color phenotypes.

I think the paper is generally clear and places the work in appropriate context. The work is based on appropriate data and sound methods (the sample sizes are a tad small, but fine given the genetic architecture of the trait). I found the interpretation of the results reasonable. As noted by the authors, there is increasing evidence in several systems that dominance and epistasis shape patterns of genetic and phenotypic variation in nature. This work nicely adds to the story and does so in a system with a tractable genetic architecture.

We sincerely thank the reviewer for positive feedback, and we appreciate constructive criticism and suggestions. We believe that by following these suggestions we substantially strengthened our inferences and improved the manuscript overall.

My main suggestions and criticisms concern the need (or benefit) of additional formal analyses to strengthen and make more quantitative some of the key results. In particular:

1. I agree that the genotype-phenotype associations presented are consistent with dominance and epistasis, but this is never formally assessed nor are coefficients of dominance and epistasis estimated. I think additional analyses to formally quantify

dominance and epistasis would really strengthen the paper. I realize that including dominance and especially all of the pairwise epistasis terms for all SNPs in the BSLMM would be intractable. But there are other options that should work. (i) You could treat (code) each of the two associated regions as a bi-allelic locus with alba and personata alleles and then fit a linear model with additive, dominance and epistasic terms to estimate the magnitude of each of these effects. Based on Figure 3, this would mostly work, though there would be some ambiguity in the encoding for a few individuals. (ii) You could do something like (i) but with each region split into ancestry blocks that show no (very little) evidence of recombination in the data set (to get around ambiguity in coding at the region/locus level). (iii) You could fit a model akin to (i) by sampling SNPs from the associated regions on 1A and 20 based on their posterior inclusion probabilities from the BSLMM (with multiple samples from the posterior you could integrate over the genotype-phenotype association posterior). (iv) You could test for marginal epistasis for each SNP in the region on 1A vs all SNPs in the region on 20 and vice a versa (you can do this with MAPIT). (v) You could re-run the BSLMM in gemma with dominance for SNPs in just the regions on 1A and 20 associated with color and for epistasis only between the SNPs with evidence of marginal epistasis. You can then ask how this affects PVE.

Thank you for detailed outline of these potential approaches to formally test for partial dominance and epistasis and to quantify their contribution to head plumage inheritance. We used the option (i) as it appears most sound given that we can avoid a problem of dealing with missing data at individual SNPs and also considering that our working hypothesis suggests that the two genomic regions at 1A and 20 effectively work as a two-locus system. To avoid the aforementioned encoding ambiguity for some highly recombinant genotypes, we excluded three personata-like individuals from this analysis. We compared a suite of models with complete and partial dominance, additive inheritance and epistatic effects using linear models. The model with patrial dominance (heterozygote appears 25% more similar to respective dominant homozygote) of personata alleles at chromosome 1A, patrial dominance of alba alleles at chromosome 20 and the epistatic term by far

*outperformed predictive power of other models. This best model (R squared = 0.894, p < 2.2e-16), revealed that the chromosome 20 term has the highest contribution to the model fit (-54.218), followed by the 1A term (-22.628) and epistatic term (15.944). Please see our new Extended data table 2, and corresponding sections of methods **L 505-515** and results **L 239-250** in the main text discussing these findings. The code and dataset for this analysis is available at https://github.com/georgysemenov/wagtails.*

2. I like the BSLMM approach, but think it is always good to assess the posterior predictive power of the model. In particular, I recommend repeating the analysis with leave-one-out cross validation to predict the phenotypes of each hybrid individual (one at a time) based on a model fit from all other individuals. One can then calculate the percent of the variation in observed phenotypes explained by the predicted phenotypes as a measure of predictive performance (this is especially useful in the context of comparisons of models with versus without dominance or epistasis; see (v) above).

*We agree that LOOCV is a good addition to our BSLMM analysis and we performed it accordingly. This analysis revealed moderate to high predictive power of the model (please see lines **135-142** and **498-503** in the main text and the new Extended data figure 3), which was consistent with other findings pointing towards non-linear relationships between phenotypic and genetic variation due to partial dominance and epistasis.*

3. The simulations add nicely to the paper, and the high variance in the outcomes across simulations is rather interesting. However, while the simulations show dominance and assortative mating can give rise to asymmetric introgression, they do not currently show that the magnitude (degree) of asymmetry is of the order seen in this data set. As these are meant (I think) to (roughly) approximate the wagtail hybrid zone, it would be nice to compare (with appropriate scaling) the magnitude of the effects seen in the simulations to what is seen in the wagtail hybrid zone.

*We agree that this is a useful comparison. We performed it by converting the simulated displacement between mating trait and neutral cline centers using post-natal dispersal distance estimated for white wagtails (Motacilla alba yarrellii) by banding (please see code and data at https://github.com/georgysemenov/wagtails/). This analysis revealed a reasonable match between asymmetry of cline displacement in simulations (358.4km across eight simulated scenarios, SD=240.8) and the observed displacement between head plumage and ancestry clines in the wagtail hybrid zone (323km, Semenov et al. 2017, Table 1). We provide these results in the main body of text (**L 266-270, 561-565**).*

Also, given that the estimate for the number of causal variants was 5.3+-2.6 (posterior SD?), it might be useful to consider simulations with two genetic regions but multiple causal variants in each. Or at least to make the choice of two loci for the simulations very clear.

*As noted above our working hypothesis suggests that the two regions on chromosomes 1A and 20 effectively act as a two-locus system, of which the region (locus) on chromosome 20 has the major effect. This working hypothesis encouraged us to run our simulations with one or two mating trait loci and we now added this rationale to methods (**L 541-545**) to better articulate our choice of simulation parameters and their relation to trait genetic architecture. We also plan to further explore genetic architecture of head plumage inheritance using long-read information for the target regions in a future, which could shed the light on the number of casual variants.*

4. I am always a bit skeptical of historical demographic inference. I would like to see that the model estimated does a good job of predicting/generating the observed data. In other words, the best parameter estimates given the general structure of the model might or might not provide an actual good fit for the data. Simulating/predicting data from the fit model can test this. Also, how confident are

you in the mutation rate used? Some indication of this and the sensitivity to the mutation rate would be nice (this always worries me as being off by an order of magnitude seems possible in many cases and would shift dates by an order of magnitude).

We thank the reviewer for his skepticism, which made us reflect on the likelihood our demographic modeling approach was recovering robust results in the face of very low genetic differentiation between subspecies, and moderately low-coverage data. Following suggestion, we used the coalescent simulator msprime to generate sequence data from our best-fit demographic model and its estimated parameter values. We simulated 100 replicates of a 10,000 bp region using the per-site mutation and recombination rate estimates presented in the previous version of the manuscript, and calculated three summary statistics to compare the fidelity of these sequences with our empirical data: 1) the joint site frequency spectrum (JSFS); 2) genome-wide pi; and 3) genome-wide dxy. We found that all three statistics from our simulated data differed significantly from the empirical data, undermining our confidence that the best-fit model was an accurate representation of the true demographic history of the subspecies.

We then decided to repeat the procedure for maximum likelihood parameter estimates of a model of strict isolation / allopatric divergence, as this scenario has the second-highest likelihood in our initial model comparison but the highest median likelihood value across all optimizations and generally more consistent results. We again found that the JSFS, genome-wide pi, and genome-wide dxy estimates were inconsistent with our sequence data (please see https://github.com/elinck/wagtails/blob/master/scripts/demographic_inference_simulations.ipynb). Additional exploratory analyses with more complex demographic models found that parameter estimates across replicate optimizations were largely unstable, suggesting that our data lack the power to distinguish among competing, biologically realistic scenarios, and / or the complexity of the true history of these populations is driving severe model misspecification.

After deliberation, we have therefore decided to cut the demographic models from the manuscript. While we understand this is a significant change to the paper at this stage in the review process, we believe it does not affect our core findings or the overall importance of our study, and believe it is preferable to presenting results with such a significant caveat. We hope the AE will understand our motivation, and we again thank the reviewer for prompting this critical re-evaluation of the analysis.

Other minor comments and suggestions (with line numbers):

L25, reword to clarify whether "local" is in geographic space or along a chromosome (I assume the latter, but not totally clear)

Done.

L27-30, this assumes minimal coupling/the lack of a strong barrier to gene flow.

We edited this sentence to reflect above points.

L61, clarify the distinction between abrupt and clinal (e.g., sharp versus gradual cline).

Changed wording accordingly.

L68-69, provide the null expectation for mating between individuals of similar phenotypes based on their proportions to put 50% in context.

For simplicity, we modified this sentence as follows: "the composition of social pairs deviated significantly from a scenario of random mating based on randomization statistical analysis[16].

L135, fd is an admixture proportion, just estimated in the context of ABBA-BABA.

We added this clarification.

L177, you could be a bit more specific about these expectations, see, e.g., Fig. 5 from https://doi.org/10.1111/eva.12380 (consider adding the triangle lines to the figure, you can almost see the points making the lines as it is).

We edited these sentences to be more specific about predictions and added a reference to the above paper. We also added triangle lines to the figure.

L194, here and elsewhere you refer to 'alba' alleles, but it is never totally clear whether you are essentially thinking of alleles at SNPs or two loci with haplotype blocks or something else. I realize this is a bit complicated, but being more specific in how you are using and thinking about this (and that you don't really know how many causal variants there are) would be nice.

*We agree with this. We now clarify this (**L 241-244**) as follows:*
"we followed an assumption of our working hypothesis that the regions on chromosomes 1A and 20 act as a two-locus bi-allelic system (although there are likely a few SNPs or haplotype blocks with key roles in influencing gene expression)."

L220, what is non-equilibrium inheritance?

Replaced with "deviation from a codominant inheritance pattern".

L302, The PCA clusters, especially on 1A really suggest a SV or other mechanism for reduced recombination.

Thank you, we agree this could be the case. Examining this possibility using the long-read technology is one of our future goals.

L428, I was a bit surprised to see such a high minor allele frequency (0.05) filter. I doubt it matters much for the paper, but is there a reason for it. Also, after filtering were genotypes called or was uncertainty in genotype taken into account?

We used a five percent cutoff to retain only relatively common variants. Genotypes were called as 0, 1, 2 following the filtering procedures.

L450, how were N's dealt with in reference, and in general unevenness in coverage across windows when calculating pi and dxy (as they divide by the number of bps in a window, which isn't really the same as the window size unless you have high coverage data across the whole window)

For the summary statistics we used the default ("coordinate") option of popgenWindows.py (https://github.com/simonhmartin/genomics_general/blob/master/README.md#diversity-and-divergence-analyses-in-sliding-windows). In this case, windows will cover a fixed range in the genome, which is defined as the window size. If there is missing data (including those in the reference genome), this can lead to variable numbers of sites used for each window. We added this clarification to the method section.

L507, not really large effects, just measurable (non-infinitesimal)

Corrected.

We would like to thank reviewer very much again for his time and helpful feedback!

Reviewer #2 (Remarks to the Author):

In "Recent hybridization and asymmetric introgression reveal the genetic architecture of a plumage trait," Semenov et al. investigate mechanisms underlying the geographic displacement of a pigmentation cline from clinal variation in genetic markers sampled from the rest of the genome in a pair of hybridizing bird subspecies. This displacement is odd because the pigmentation trait contributes to assortative mating between the subspecies, which should give rise to concordant clines. To explain this seemingly counterintuitive pattern (one that is seen in other hybrid zones as well), they sequenced whole genomes of phenotypically diverse individuals sampled from the hybrid zone plus allopatric parental populations. With these data, they examined patterns of genomic differentiation and genotype-phenotype association and found two regions that had both the highest levels of differentiation and strongest association with plumage phenotypes. Based on these analyses and on a BSLMM
analysis that enabled them to estimate parameters related to genetic architecture (e.g., PVE and # of loci with "sparse" effects), they concluded that pigmentation differences were attributable to a simple genetic architecture. Next, they examined ancestry in these two regions in hybrids that fell into different phenotypic categories (e.g., parental-like, intermediate, etc.) to make inferences about dominance and epistasis. Using the inferred inheritance model, they simulated pigmentation and genomic clines under a variety of conditions to test the hypothesis that observed trait inheritance could explain the displacement of clines. Although cline movement appeared to be highly stochastic across simulations, they did find that when pigmentation clines were displaced, they had a strong tendency to be displaced towards the dominant homozygote, which is consistent with the observed cline. Finally, to put this cline in the proper historical context, they estimated demographic parameters under a model of secondary contact and found that the timing of hybridization coincides with anthropogenic changes to the habitat. Overall, these results contribute to our understanding of how trait genetic architecture can give rise to displaced clines, even for traits that mediate assortative mating. They also

provide a likely empirical example of human-mediated hybridization.

This paper was an absolute pleasure to read. It is very well written, interesting, and addresses an important conundrum in evolutionary biology. There is a substantial amount of data included in the paper, the analyses are appropriate and reflect current best practices (to the best of my knowledge), the results are clearly communicated and interpreted, and the major conclusion of the paper--that the displacement of the pigmentation cline can be explained by trait genetic architecture--is supported by the data. The authors are also clear about the limitations of the study, acknowledging that cline movement is highly stochastic and other mechanisms could potentially explain the observed clines. I also like that the authors did not bog down the paper with a bunch of extraneous analyses and results that were unrelated to the central questions of the paper. I rarely encounter a paper that I think can be published as is, but I think this is one of those rare exceptions. And I am
confident that these results would be of broad interest to readers of Nature Communications. Thanks to the authors for making my job as a reviewer so easy!

We sincerely thank the reviewer for such a positive assessment of our manuscript!

I had one minor comment:

L67-69: “Strong positive assortative mating by head plumage (~50% of pairs are formed between the same parental phenotypes)…” I read this line several times and am still confused. If 50% of pairs are same-phenotype, doesn’t that mean that 50% of pairs are different-phenotype? That seems like random mating. Did the authors mean something else here? Maybe same-phenotype pairs are 50% more common? Please reword for clarity or explain how this is strong assortative mating.

We agree that this phrasing was misleading (also pointed out by the reviewer #1). In our previous study we refer to, we used randomization (bootstrap) statistics to

assess whether observed pair composition deviates from a scenario of random mating. We replaced the above phrasing with following:

"Strong positive assortative mating by head plumage (i.e., the composition of social pairs deviated significantly from a scenario of random mating based on randomization statistical analysis[16]) and the observed predominance of parental phenotypes over hybrids are consistent with the maintenance of restricted introgression of head plumage by selection."

We once again would like to thank the reviewer for their time in reviewing our manuscript and for positive evaluation of our work!

Reviewer #1 (Remarks to the Author):

All of my comments were adequately and thoughtfully addressed, and I am very much happy with the current version of the manuscript. I fully support the authors' decision to remove the demographic inference section and I think that the paper is actually stronger and more focused without it.

Zach Gompert
